# Cytoplasmic Male Sterility Contributes to Hybrid Incompatibility Between Subspecies of *Arabidopsis lyrata*

**DOI:** 10.1534/g3.113.007815

**Published:** 2013-10-01

**Authors:** Esa A. Aalto, Hans-Peter Koelewijn, Outi Savolainen

**Affiliations:** * Department of Biology, University of Oulu, FIN-90014 Oulu, Finland; † Biocenter Oulu, University of Oulu, FIN-90014 Oulu, Finland; ‡ Nunhems Netherlands B.V., 6080 AA Haelen, the Netherlands

**Keywords:** *Arabidopsis*, cytoplasmic male sterility, hybrid incompatibility, QTL mapping, speciation

## Abstract

In crosses between evolutionarily diverged populations, genomic incompatibilities may result in sterile hybrids, indicating evolution of reproductive isolation. In several plant families, crosses within a population can also lead to male sterile progeny because of conflict between the maternally and biparentally inherited genomes. We examined hybrid fertility between subspecies of the perennial outcrossing self-incompatible Lyrate rockcress (*Arabidopsis lyrata*) in large reciprocal F2 progenies and three generations of backcrosses. In one of the reciprocal F2 progenies, almost one-fourth of the plants were male-sterile. Correspondingly, almost one-half of the plants in one of the four reciprocal backcross progenies expressed male sterility. In an additional four independent F2 and backcross families, three segregated male sterility. The observed asymmetrical hybrid incompatibility is attributable to male sterility factors in one cytoplasm, for which the other population lacks effective fertility restorers. Genotyping of 96 molecular markers and quantitative trait locus mapping revealed that only 60% of the plants having the male sterile cytoplasm and lacking the corresponding restorers were phenotypically male-sterile. Genotyping data showed that there is only one restorer locus, which mapped to a 600-kb interval at the top of chromosome 2 in a region containing a cluster of pentatricopeptide repeat genes. Male fertility showed no trade-off with seed production. We discuss the role of cytoplasm and genomic conflict in incipient speciation and conclude that cytoplasmic male sterility–lowering hybrid fitness is a transient effect with limited potential to form permanent reproductive barriers between diverged populations of hermaphrodite self-incompatible species.

During allopatric isolation of populations, genic incompatibilities reducing hybrid fitness (Bateson-Dobzhansky-Muller incompatibilities) can evolve (Bateson 1909; Dobzhansky 1936; Muller 1939). In plants, hybrid sterility, especially hybrid male sterility, is the most common postmating reproductive barrier (Lowry *et al.* 2008), even if other barriers also contribute to isolation (Bomblies and Weigel 2007; Bomblies and Weigel 2010; Lexer and Widmer 2008). Detailed mechanisms of nuclear incompatibilities have rarely been studied (Mizuta *et al.* 2010; Moyle and Graham 2005; Sweigart *et al.* 2006). Symmetric incompatibilities are mostly attributable to interactions between nuclear loci, but some asymmetric incompatibilities can also have a nuclear origin, for instance, in case of interactions between dominant and recessive alleles (Sweigart *et al.* 2006). In contrast, nuclear cytoplasmic interactions are specifically asymmetric incompatibilities in which the phenotype depends on the cytoplasm of the hybrid (*i.e.*, maternal parent of the initial cross).

Darwin already noticed that hybrids raised from reciprocal crosses generally differ in their degree of sterility (Darwin 1859). More recently, Tiffin *et al.* (2001) observed significant asymmetries in reproductive isolation of all three Angiosperm genera they studied. Such asymmetry is often attributable to cytonuclear incompatibilities (Rieseberg and Blackman 2010). Indeed, 35% of 23 angiosperm species pairs (both hermaphrodite and dioecious) tested showed asymmetrical differences in hybrid pollen viability, indicating possible differentiation of cytoplasmic male sterility (CMS) factors between the species (Tiffin *et al.* 2001). However, asymmetric postmating isolation in reciprocal crosses may also be attributable to other factors, such as genetic maternal effects, and in flowering plants it may be attributable to gametophytic–sporophytic or triploid endosperm interactions, where haploid paternal and diploid maternal genomes interact (Turelli and Moyle 2007).

The CMS genes in mitochondria cause male sterility in plants by interfering with the mitochondrial respiratory system (Chase 2007). Male fertility can be restored by fertility restorer (*Rf*) genes located in the nucleus. Both the CMS and *Rf* genes have been identified in a number of crop plants (Hanson and Bentolila 2004; Yamamoto *et al.* 2008) and in *Mimulus* (Barr and Fishman 2010; Case and Willis 2008). In most cases, CMS is caused by rearrangements of mitochondrial open reading frames (ORFs). These novel chimeric genes have a negative impact on the male phenotype but do not reduce transmission of the maternally inherited mitochondrial genome (Case and Willis 2008; Chase 2007; Hanson and Bentolila 2004). The known fertility restorers are mostly members of a class of pentatricopeptide repeat (PPR)–containing genes that modify transcripts in mitochondria and thus are capable of evolving to silence male sterility–causing ORFs (Bentolila *et al.* 2002).

The population genetics of CMS has been most extensively studied in natural gynodioecious populations. CMS spreads because plant nuclear genomes are transmitted to following generations via pollen and ovule, but cytoplasmic genomes, mtDNA and cpDNA, most often are transmitted by the ovule only. This leads to a possible genomic conflict (Cosmides and Tooby 1981), because a maternally inherited male sterility gene can invade a hermaphrodite population. Selection may even favor the invasion if female plants have increased female fertility relative to hermaphrodites, because of reduced energetic investment in male floral organs or because hermaphrodites may suffer inbreeding depression caused by selfing. (Charlesworth and Ganders 1979; Dufay and Billard 2012; Hanson and Bentolila 2004; Shykoff *et al.* 2003). However, theoretical studies show that CMS can become fixed even when females have the same ovule production as hermaphrodites (Charlesworth and Charlesworth 1978; Gouyon *et al.* 1991). Theory predicts rapid fixation of both CMS genes and the corresponding fertility-restoring alleles, which means that populations can be carriers of different CMS and matched restorers despite being hermaphroditic. However, balancing selection can maintain polymorphisms of several CMS-restorer systems in gynodioecious populations (Charlesworth and Ganders 1979; Charlesworth 1981; Frank 1989).

Most of the work regarding the genetic basis of cytonuclear sterility has been in such polymorphic gynodioecious species (Koelewijn and van Damme 1995b). In one of the first studies, several CMS-restorer systems were found in *Origanum vulgare* (Kheyr-Pour 1981). Later, Koelewijn and van Damme (1995a; Koelewijn and van Damme 1995b; Koelewijn 2003; van Damme *et al.* 2004) found four types of CMS, each having multiple restorers in *Plantago coronopus*. The genetics of CMS has been resolved best in *P. coronopus* and *Plantago lanceolata* (DeHaan *et al.* 1997; van Damme and van Delden 1982). In many cases, each CMS-causing factor is restored by a single restorer gene, but in *Silene* and *Thymus*, epistatic interactions of multiple restorer genes are needed to explain the observed female hermaphrodite ratios (Belhassen *et al.* 1991; Charlesworth and Laporte 1998; Garraud *et al.* 2011).

The genetic basis of male sterility also has been widely studied in crop plants, especially in rice, in which several nuclear genic male sterility (NMS) and CMS–*Rf* systems have been discovered (Li *et al.* 2007) and the molecular basis of some of them has been already resolved (Ding *et al.* 2012; Long *et al.* 2008; Mizuta *et al.* 2010; Wang *et al.* 2006; Yamagata *et al.* 2010). These discoveries are important in developing new crop varieties and understanding molecular mechanisms of male sterility, but they do not inform about natural dynamics of CMS (and NMS) in hermaphrodite plant populations.

NMS factors have been described in *Mimulus*, in which a dominant *Mimulus guttatus* allele at the *hybrid male sterility 1* (*hms1*) locus acts in combination with recessive *Mimulus nasutus* alleles at *hms2*, causing nearly complete male sterility in hybrids (Sweigart *et al.* 2006). In addition to CMS, NMS, and seed infertility, a further signal of genomic incompatibility is transmission ratio distortion (TRD), often found in crosses between diverged species or populations (Fishman *et al.* 2001; Hall and Willis 2005; Harushima *et al.* 2001; Phadnis 2011).

Yet, we do not know much about the genetic basis and variability of CMS in wild hermaphrodite populations and its possible role in reproductive isolation. In the current study, we focus on that question while examining hybrid sterility in a cross between subspecies of the perennial outcrossing self-incompatible plant Lyrate rockcress (*Arabidopsis lyrata*) (O’Kane and Al-Shehbaz 1997; Savolainen and Kuittinen 2011). The subspecies *A. l. lyrata* and *A. l. petraea* (Balañá-Alcaide *et al.* 2006; Muller *et al.* 2008; Ross-Ibarra *et al.* 2008) diverged approximately 300,000 years ago and are highly differentiated (K_s_ = 0.04) (Pyhäjärvi *et al.* 2012), thus providing a suitable model system for resolving the questions related to early-stage speciation. *A. lyrata* has diverged from its well-studied close relatives *A. thaliana* and *A. halleri* approximately 10 million years ago and 1.3 million years ago, respectively (Ossowski *et al.* 2010; Pyhäjärvi *et al.* 2012; Roux *et al.* 2011). In addition, a reference genome is available for the ssp. *lyrata* (Hu *et al.* 2011).

Despite the high divergence, crosses between the subspecies of *A. lyrata* produce viable progeny without any apparent difficulties. However, there were a considerable number of transmission ratio–distorted loci in a cross between populations from Sweden and Russia (Kuittinen *et al.* 2004) and between a North Carolinian and Norwegian population (Leppälä and Savolainen 2011). Further, in the latter pair, hybrid male fitness was reduced because of supposed nuclear genic incompatibilities and a putative CMS. Quantitative trait loci (QTL) mapping detected one locus restoring fertility, which was located at the top of chromosome 2. Another hidden locus for fertility restoration was suspected. In addition, some evidence for pollen viability and seed production QTL was found (Leppälä and Savolainen 2011).

To characterize in more depth the genetics of the incipient reproductive isolation between the subspecies of *A. lyrata*, we have conducted large crossing experiments between the Norwegian and North Carolinian populations. We first confirmed that there is a CMS system in this cross and then showed how it is inherited. To infer within-population dynamics of CMS, we searched for polymorphisms in CMS or restorers in either of the populations. We mapped the fertility restorer of the CMS at the top of chromosome 2 with a dense set of markers near the previously identified putative restorer QTL. In this large cross, with more power than in the study by Leppälä and Savolainen (2011), we searched for the other putative QTL for fertility. We also examined TRD across the genome and its impact on the genetic analyses and inferences on the number of genes involved in restoration. Finally, we herein discuss the dynamics of CMS in isolated populations and its role as a potential reproductive barrier between distantly related populations of hermaphroditic species.

## Materials and Methods

### Populations

We studied two populations of *A. lyrata*, representing its two subspecies. The European population represents ssp. *petraea* and is from Norway [Spiterstulen, abbreviated hereafter as Sp (61°38′N, 8°24′E)] and the North American population represents ssp. *lyrata* from North Carolina [Mayodan, hereafter abbreviated as Ma (36°2′N, 79°58′W)]. Silent site nucleotide divergence between these populations is approximately 4.3%, F_ST_ based on synonymous SNP data of 19 genes is 0.629, and the estimated divergence time is approximately 140,000 generations (Pyhäjärvi *et al.* 2012).

### Crosses for hybrids: F1, F2, and pseudobackcrosses

#### F1:

We produced eight families of F1 plants to compare them with F2 plants to find dominance effects. These F1 plants were also used to produce eight families of F2 plants for examining polymorphism of CMS in the study populations. The starting material for the F1 families were four full-sib families from Sp and four full-sib families from Ma, which Leppälä and Savolainen (2011) used as parental controls and crossed with Ma and Sp pollen donors to produce the F1 plants. Seeds from these crosses produced 565 F1 plants (244 SpMaF1 and 321 MaSpF1 having four cytoplasms sampled from the wild Ma and four cytoplasms sampled from the wild Sp population, respectively) that were grown during the 2007 experiment (presented in Supporting Information, Figure S1 in the middle and on the right with yellow and blue backgrounds).

#### F2:

**Figure 1:**
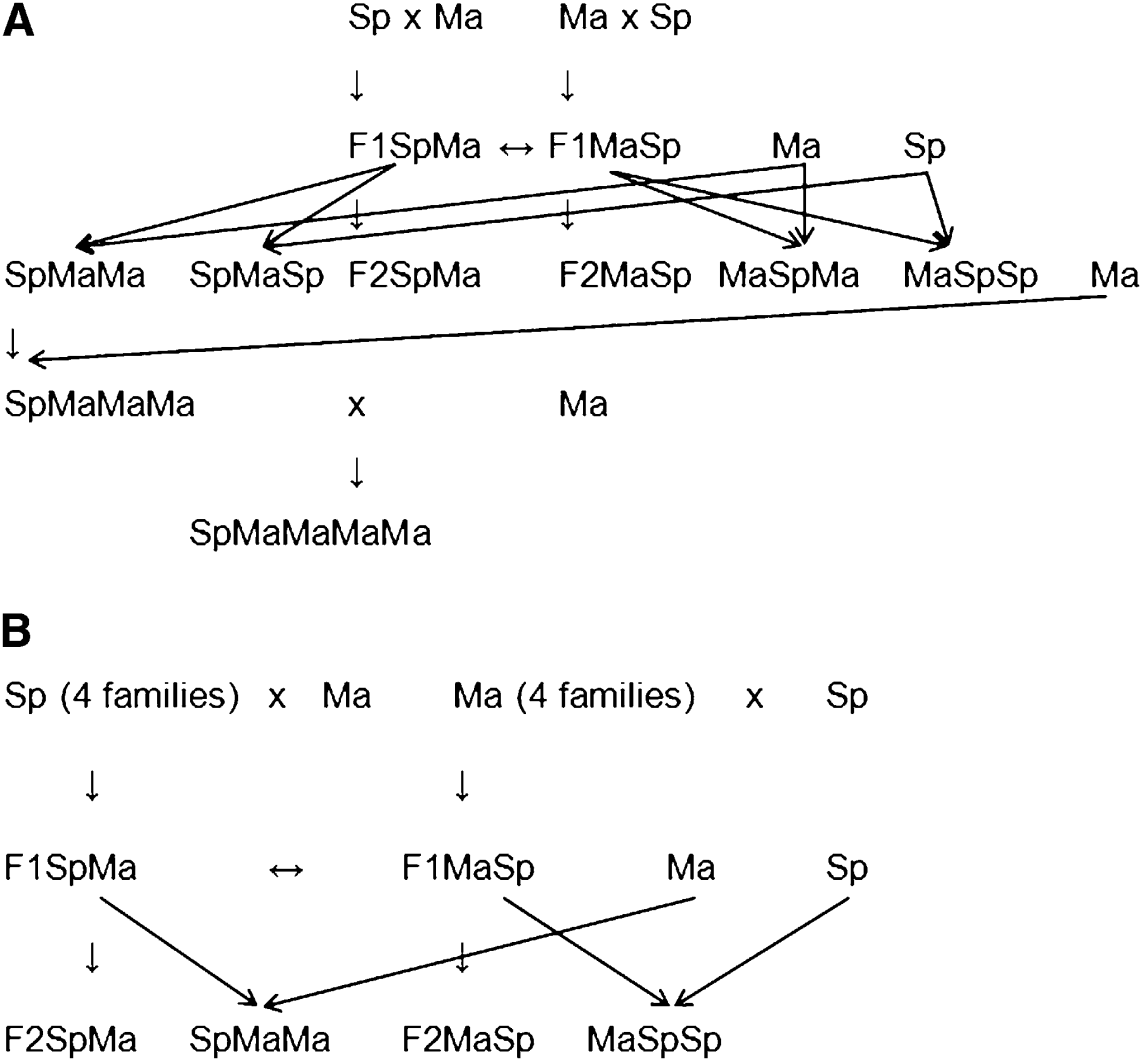
Simplified crossing schemes. × (or just arrow) = one-way cross. ↔ = two-way cross. Arrows indicate progeny of the plant. Detailed crossing scheme is presented in Figure S1. (A) The main crossing family. (B) Crosses to examine cytoplasmic male sterility (CMS) polymorphism.

We used unrelated plants in all crossing designs to avoid expression of self-incompatibility and inbreeding depression. In 2007, we grew a large F2 family (745 SpMaF2 with Sp cytoplasm and 1352 MaSpF2 with Ma cytoplasm; presented in Figure S1 on the left with green background; simplified presentation available in Figure 1A) to get precise estimates of male fertility and seed production per silique of these hybrids, to compare results with those of a previous study (Leppälä and Savolainen 2011), and to create a comprehensive genetic linkage map. The 2007 large F2 families were full sisters of the plants used in the previous study (Leppälä and Savolainen 2011). The parents of this cross were grown from field-collected seeds. One Sp pollen donor was crossed to the Ma pollen recipient to produce F1 hybrids with Ma cytoplasm, and another pair of Ma and Sp plants was crossed reciprocally to produce F1 hybrids with Sp cytoplasm (in the upper left corner with green background in Figure S1). Two of the F1 hybrids were then crossed reciprocally to yield two sets of F2 individuals with different cytoplasms.

#### Backcrosses:

To obtain unequivocal results on whether asymmetry between reciprocal F2 plants is attributable to cytoplasmic factors, and to examine the genetics, we generated four pseudobackcross populations, [MaSp]Sp (188 plants), [MaSp]Ma (259 plants), [SpMa]Sp (82 plants), and [SpMa]Ma (116 plants), grown during the 2008 experiment (middle left on green background in Figure S1). These are pseudobackcrosses because they are structured like a backcross, but we could not use the same plants or closely genetically related individuals in the same pedigree because of the outcrossing nature of our study species (Figure 1A, Figure S1). To do so, we used F1 plants, full sibs of the parents of the year 2007 F2 progeny (F1 plants on the left with green background in Figure S1) as maternal parents for the first-generation pseudobackcrosses (BC1). These 14 MaSpF1 and nine SpMaF1 plants were pollen recipients in a cross with one Ma and one Sp pollen donor (crosses performed by Leppälä and Savolainen 2011).

#### Advanced pseudobackcrosses to solve the detailed genetic basis of CMS:

To get a more detailed view of the genetics of asymmetric male sterility, we continued backcrossing past BC1. In total, we had 1389 of (SpMa)MaMa pseudobackcross plants during the 2008 experiment (Figure 1A and middle left with green background in Figure S1), with a total of 91 mothers and 8 grandmothers that were all full sister F1 plants (SpMaF1). Because the CMS appeared only in the [SpMa]Ma (and SpMaF2) plants, we chose to grow only those second-generation backcross plants (BC2) that had cytoplasm from Sp and seven-eighths of the nuclear genome from Ma. The pollen donors used to create these second-generation pseudobackcrosses were cloned tissue-cultured plants, two Sp clones and two Ma clones.

To further examine the inheritance of the CMS and to find possible polymorphism in Ma restorers, we produced a third-generation pseudobackcross population with multiple Ma pollen donors. We grew 745 (SpMa)MaMaMa plants (hereafter referred to as BC3) with cytoplasm from Sp and 15 of 16 nuclear genomes from Ma in 2009 (lower left on green background in Figure S1). These are progeny of 37 BC2 plants crossed with 12 Ma pollen donors. The donors were from four unrelated seed families that were Ma plants grown from seeds produced in laboratory crosses for two generations after collection from the field.

#### Examining polymorphisms of CMS:

To examine possible within-population variation in CMS, we generated several F2 and backcross families. We obtained progeny of 562 F2 [341 SpMaF2 and 221 MaSpF2 offspring of 113 parental F1 plants (81 mothers, 74 fathers)] from 7 (4 Sp and 3 Ma) maternal lineages. The starting material for the F2 progenies were the F1s described, which were crossed reciprocally with other F1 plants in a randomized block experimental design (two arrowhead lines in the middle of Figure S1). The cytoplasms of these lineages were not related more than expected by chance in wild populations. We obtained 287 (SpMa)Ma pseudobackcross plants having 4 Sp cytoplasms and three-fourths of the nuclear genome from Mayodan and 364 reciprocal (MaSp)Sp plants having 4 Ma cytoplasms and three-fourths of the nuclear genome from Spiterstulen. They were produced from the F1 as F2 plants described by using the F1 as pollen recipients in crosses with the cloned tissue-cultured plants (one Sp clone and one Ma clone). These same clones also had been used as pollen donors in the second-generation backcrosses described. These F1 and BC families were grown in 2008 and are presented in Figure 1B and in Figure S1 on blue (Sp cytoplasm) and yellow (Ma cytoplasm) backgrounds.

### Greenhouse experiments

#### Growth conditions and plants:

The growth conditions used are those described previously (Leppälä and Savolainen 2011). Briefly, seeds were germinated on cell culture dishes, planted in pots in fully randomized order, grown in the greenhouse for 4 wk, vernalized in cold room at +4° for 8 wk, and, finally, grown to flowering in the greenhouse for long days (20 hr) under natural midsummer light and temperatures. Biological pest control was used to reduce damage caused by pest insects and plant diseases. In each experiment, we had population plants from several seed families of Sp and Ma to control for possible variation between blocks and years. All the crosses are summarized schematically in Figure S1 and in a simplified way in Figure 1.

#### Scoring fertility:

To assess male fertility, we measured quantity and quality of pollen of the plants. Two unopened buds were collected from each plant and anthers were dissected and immediately crushed and mixed with 25 μl lactophenol-aniline blue staining solution (Kearns and Inouye 1993). For most of the plants, we collected two of the first buds after the first flower (buds two through five), but in some cases later buds were used. We tested whether the number of the bud collected affects pollen number or quality and did not find any correlations. Samples were stored in cold and dark conditions. We counted the numbers of viable (stained) and inviable (nonstained) pollen grains from a subsample of 0.075 µl in a hemocytometer under a microscope. From these data, we calculated the average number of all pollen grains and the proportion of viable pollen grains in the sample. We used these values as the measure of male fertility of the plant because they are directly proportional to the total number of pollen grains in the flower and the proportion of the viable pollen grains. There was a discontinuity in the cumulative distribution of the number of pollen grains at approximately 10 pollen grains (Figure S2). Therefore, the plants that produced less than 10 pollen grains in the counted subsample were considered male-sterile and the proportion of viable pollen grains was not calculated for them. The average number of pollen grains counted per control sample increased from the 2007 experiments to 2009 experiments, likely because of improvements in growing conditions and more careful handling of the samples. However, this had no noticeable effect on the number of samples with low pollen number, because the discontinuity at approximately 10 pollen grains did not shift upward (Figure S2), allowing us to compare results between years without any adjustments.

In addition, we scored the morphology of the anthers during the 2008 experiments simply by classifying them as “normal” or “without pollen,” which was usually associated with other developmental abnormalities as well.

As other fertility-associated fitness traits, we measured seed quantity and quality in the 2007 experiment. To obtain seeds, we pollinated two flowers from each plant with Sp and another two with Ma pollen. Pollen donors were two tissue-cultured plants from each population. We collected siliques when mature and counted the seeds. Seeds were classified as being of good quality (plump) or of low quality (shriveled or green). As overall fitness measures, we measured rosette sizes of the plants and counted the number of flowering shoots produced during the flowering season.

#### Data analysis:

Because none of the variables we measured was normally distributed, we tested differences in male and female fertility between parental, F1, F2, and backcross plants and between different families for significance with Kruskal–Wallis rank-sum test, which is a nonparametric equivalent of the one-way ANOVA, appropriate for non-normally distributed data. When the Kruskal–Wallis test suggested significant differences, we identified the significant pairwise comparisons using Wilcoxon rank-sum tests. Statistical tests were performed with R 2.9.0 (R Development Core Team 2010).

### DNA analyses

#### Genotyping:

For genotyping, we collected leaves from the plants at the end of the experiment and extracted DNA in plate format with EZ-96 Plant DNA kit (Omega Bio-tec) with an automatic pipetting robot (Microlab STAR; Hamilton Robotics). We genotyped the four parental, two F1, and 1880 F2 plants of the experiment performed in 2007. The plants were genotyped for 96 SNP markers in 75 genes chosen to cover the genome with at least 10–20 cM of distance between them. The marker density was set higher at the top of chromosome 2 to fine-map *Rf*. Genotyping was performed at Finnish Institute of Molecular Medicine using the MALDI-TOF method (Sequenom). We obtained 98.4% of the marker genotypes for all individuals; the rest were missing for technical reasons. To get more informative markers, we combined the information of SNPs located in the same gene. All genotype information and phenotype information are included in supporting information File S1, File S2, File S3, and File S4.

#### QTL-mapping fertility:

Genetic linkage maps were generated using CRI-MAP (Green *et al.* 1990) version 2.503. The linkage groups and the order of markers in them corresponded exactly to what was expected based on the physical genomic data of *A. lyrata* (Hu *et al.* 2011). To find possible genotyping errors, we used the “chrompic” option of CRI-MAP. None of the markers showed unusually high double-recombination frequency, but 20 individuals (1%) had exceptionally many (5–13) double-recombinations and were removed from the final analysis. All map distances are expressed as Kosambi cM.

We performed QTL analyses with R/qtl (Broman *et al.* 2003). None of the measured phenotypes was normally distributed and we could not find suitable transformation for them; thus, we used nonparametric interval mapping for all traits. QTL analyses were conducted separately for the SpMaF2 and reciprocal MaSpF2 to detect possible maternal (cytoplasmic) effects.

Genetic incompatibilities typically involve two or more loci. Therefore, we performed single-locus QTL scans followed by two QTL scans. The two-QTL model can also detect epistatic interactions between two loci possibly involved in BDM incompatibilities or CMS systems with several restorers. For the QTL detected, we estimated additive, dominance, and parent-of-origin effects. The genome-wide threshold for significance of QTL was set by permutations (n = 1000).

We inspected transmission of each genotype of each marker separately and calculated Bonferroni-corrected significances for comparing the observed ratios to equal transmission of the genotypes.

## Results

### Reduced pollen number and pollen viability in F1 and F2

**Figure 2:**
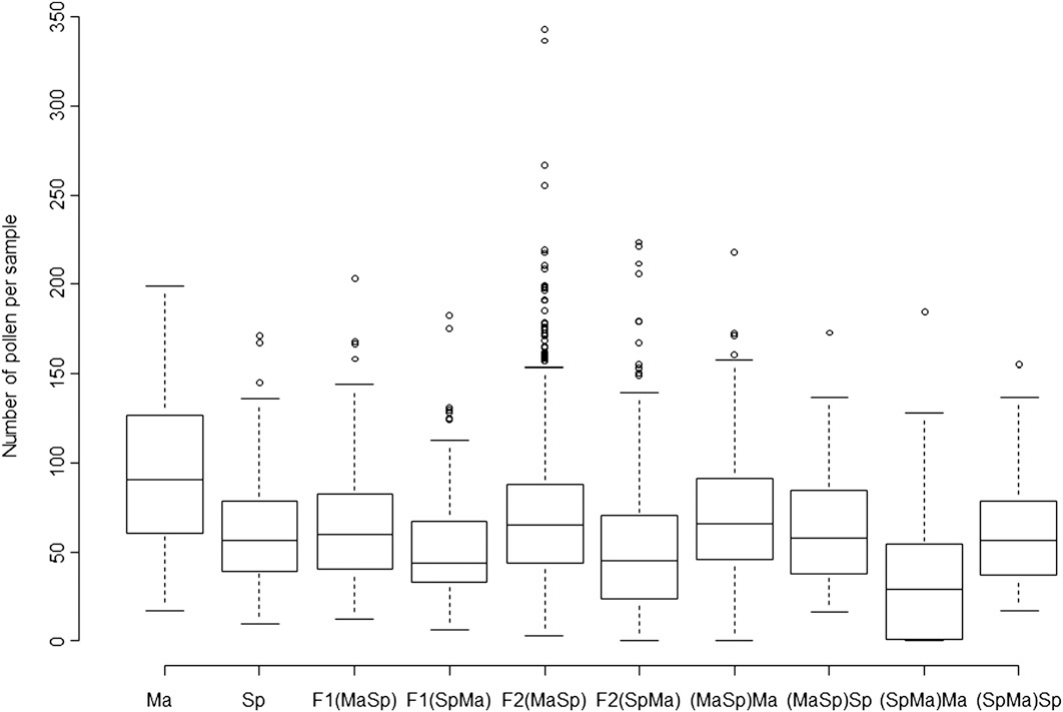
Pollen number summary of the 2007 crossing experiment including reciprocal F2s and backcrosses belonging to the main crossing family. Number of pollen per sample (horizontal line, median; box, quartiles; dots, outliers) for parental populations, F1 and F2 hybrid reciprocals, and four types of backcrosses. F1 and F2 hybrids with Spiterstulen (Sp) cytoplasm produce less pollen than their reciprocals. In backcrosses, MS plants appeared only within (SpMa)Ma progeny.

The two parental populations were highly male-fertile. In the three experiments performed in 2007 (F1, F2, and BC1), 2008 (BC2 and several F2 and BC1 families), and 2009 (BC3), the medians of average pollen production of the Sp and Ma plants were 75.9 and 109.6 pollen grains per sample, respectively. Corresponding pollen fertilities (pollen dyed blue in aniline blue staining) were 91.4% for Sp and 92.6% for Ma. The fertility of the F1 was reduced compared with the parental populations, and there was a significant (*P* < 0.01) difference in pollen numbers between the reciprocals, plants with Ma cytoplasm producing more pollen (Figure 2).

**Figure 3:**
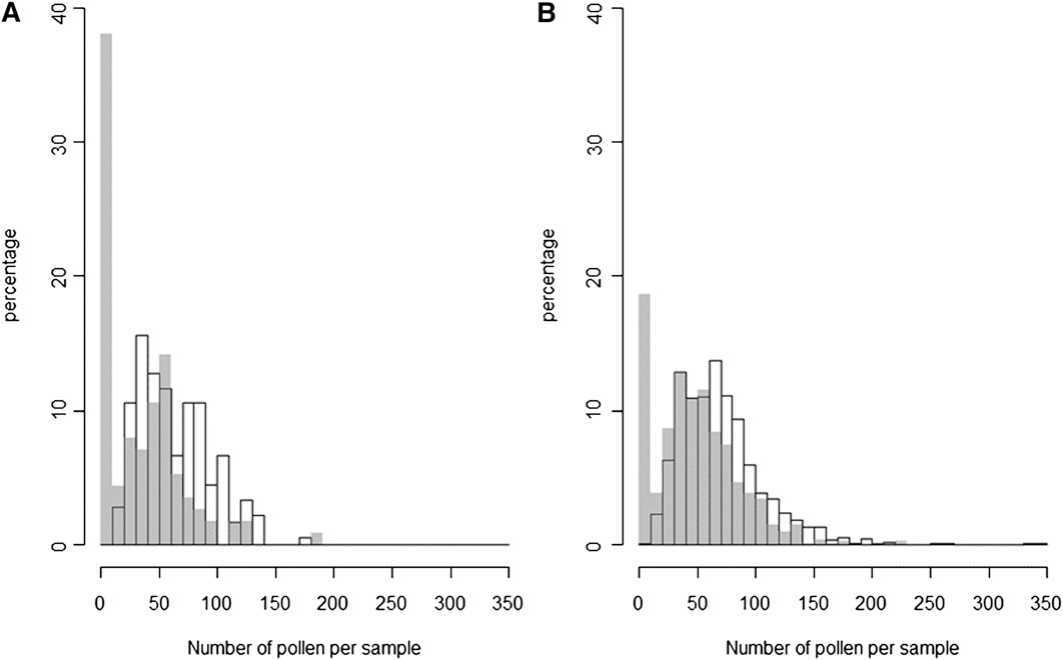
Cytoplasm affects pollen production of *A. lyrata* hybrids. Plants with Spiterstulen (Sp) cytoplasm (gray shading) produced less pollen than plants having Mayodan (Ma) cytoplasm (black borders). (A) Reciprocal backcross hybrids (SpMa)Ma and (MaSp)Sp. (B) F2 hybrids.

In the F2 generation there was again a significant difference in pollen numbers (*P* < 0.01) between plants with Ma and Sp cytoplasms, with Ma cytoplasm leading to 40% higher pollen production; however, average pollen number and fertility of neither reciprocal differed from the corresponding F1 generation (Figure 2, Figure S3). As expected, the variance in pollen number was much higher within F2 than F1 plants (*P* < 0.001). Slightly less than one-quarter, 20.7%, of SpMaF2 plants were male sterile (MS), *i.e.*, produced very little or no pollen, but in MaSpF2 as well as in F1 only 1.1% plants were MS (Figure 2, Figure 3A, Figure S3). When excluding the MS plants, the pollen number per sample produced by SpMaF2 was still lower than that of MaSpF2 (59.7 and 70.3, respectively; *P* < 0.001).

### Backcrosses show that asymmetric reduced fertility is attributable to CMS

Asymmetric hybrid sterility can occur for multiple reasons, but here we show that in our case CMS was the cause. Assuming a CMS model with one dominant major fertility restorer, we would expect that MS plants appear also in one of the backcrosses, namely [SpMa]Ma, which indeed was the case: 37.2% of 113 [SpMa]Ma plants that flowered were MS (Table 1). The mean pollen numbers per sample and pollen fertilities of hermaphrodites (H) varied between the four different backcrosses [MaSp]Ma, [MaSp]Sp, [SpMa]Ma, and [SpMa]Sp (Figure 2, Figure 3B, Figure S3). A Kruskal–Wallis test detected highly significant (*P* < 0.001) differences between groups in both measures due to lower pollen number and fertility of [SpMa]Ma compared with all the others and no differences in the other pairwise comparisons. This also was the case when backcrosses were compared with the parental populations. Compared to Ma the pollen numbers and fertilities were always lower, but compared to Sp only [SpMa]Ma plants differed significantly (in both measures) (Figure 2, Figure 3B, Figure S3). Thus, male sterility segregates only in crosses in which the progeny has cytoplasm from Sp and nuclear Ma alleles from both parents (Table 1).

**Table 1:** Summary of observed H:MS ratios of the main crossing family (single Ma and Sp cytoplasm involved)

Cross	2007		2008		2009		Leppälä and Savolainen (2011)		Best Fitted Ratio
	H	MS	H	MS	H	MS	H	MS	
**Base**									
**Ma × Ma**	117	0	109	0					1:0
**Sp × Sp**	126	1	66	2					1:0
**F1**									
**Ma × Sp**	305	0							1:0
**Sp × Ma**	233	4							1:0
**F2**									
**MaSp × SpMa**	1303	14					180	0	1:0
**SpMa × MaSp**	550	144					165	25	13:3
**BC1**									
**SpMa × Ma**	71	42	116	58					5:3
**SpMa × Sp**	71	4							1:0
**MaSp × Ma**	254	2							1:0
**MaSp × Sp**	180	0	151	1					1:0
**BC2**									
**(SpMa)Ma × Ma**									
**H plants**			528	503					1:1
**MS plants**			50	199					3:13
**BC3**									
**(SpMa)MaMa × Ma**									
**H plants**					289	178			5:3
**MS plants**					44	198			3:13

There is H:MS segregation in one of the reciprocal F2s and in backcrosses with Sp cytoplasm when crossed with Ma population, providing strong evidence of cytoplasmic effect on male sterility. H, hermaphrodite; MS, male-sterile; Ma, Mayodan; Sp, Spiterstulen.

At first sight, the H:MS ratios may seem to fit with a two-restorer loci model (one dominant plus one recessive; H:MS ratio in F2 = 13:3); however, when further crossing generations are taken into account, the data do not support this hypothesis because we do not see expected segregation of putative recessive restorer there (Table 1).

**Figure 4:**
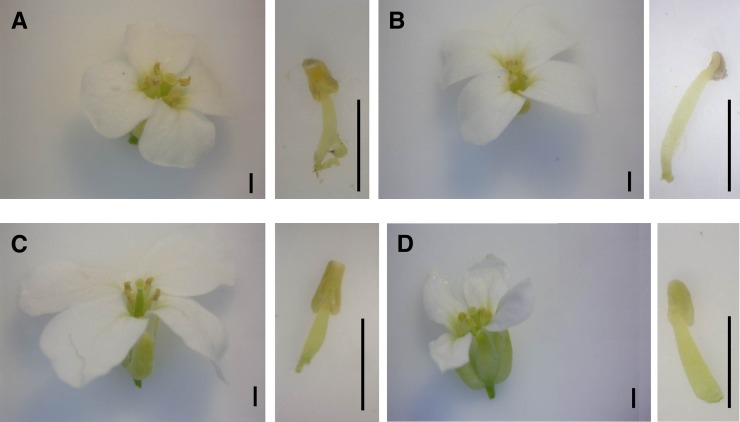
Flower and anther morphology. Flowers and anthers of Spiterstulenian (A), North Carolinan (B), and two (SpMa)MaMa second-generation backcross hybrid (C, D) plants. Some hybrids have fully developed flowers and anthers (C), whereas others develop smaller flowers with malformed petals and pollenless anthers (D). Scale bars are 1 mm.

To find out visual appearance on male-sterile anthers, we examined correlation of anther phenotypes with CMS. The phenotypes ranged from MS plants having anthers almost totally absent to H plants having fully developed anthers covered with yellow pollen (Figure 4). The appearance of the anthers was highly correlated with the estimated pollen numbers (Figure S4).

### Mapping revealed only one *Rf* of CMS

**Figure 5:**
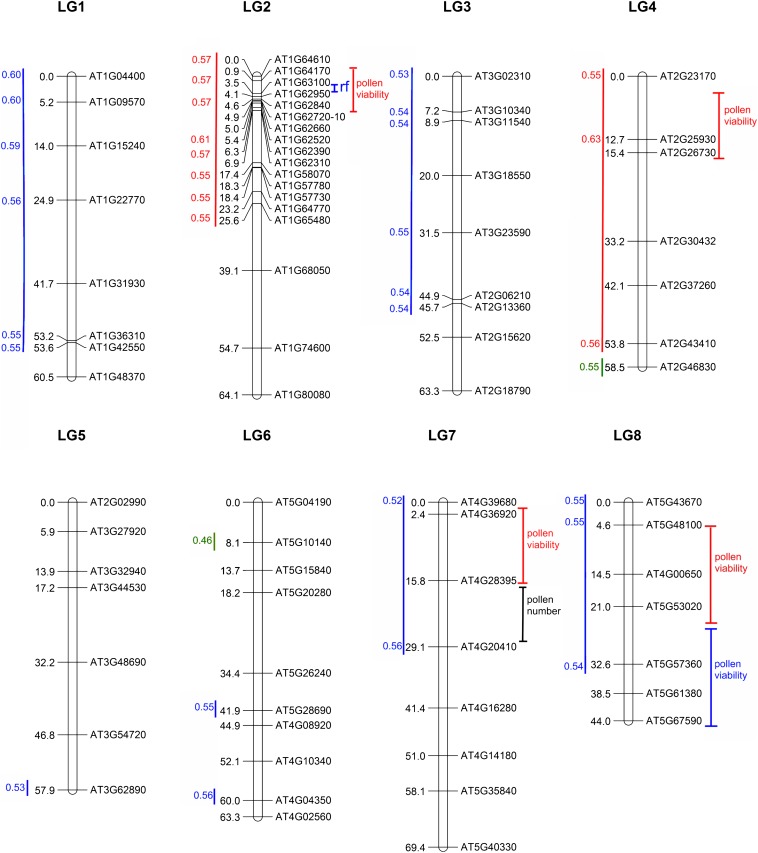
Transmission ratio distortions (TRD) and quantitative trait loci (QTL) for male fitness–related traits projected on the linkage map of *A. lyrata* ssp. *petraea* Spiterstulen (Sp) × *A. lyrata* ssp. *lyrata* Mayodan (Ma). Marker positions are indicated by horizontal lines with distances in cM on the left and gene ID numbers on the right. TRD is presented on the left side of each chromosome. Blue lines indicate favored Sp alleles and red lines favored Ma alleles. Numbers show Sp and Ma allele frequencies. Green lines present unexpected number of heterozygotes and numbers show heterozygote frequencies. Values for low-informativeness markers are not shown. QTL with 95% confidence intervals for restorer of fertility (*rf*) and pollen quantity and viability are shown on the right of each chromosome. Cytoplasm-dependent QTL are indicated by colors (blue = Sp; red = Ma cytoplasm).

**Figure 6:**
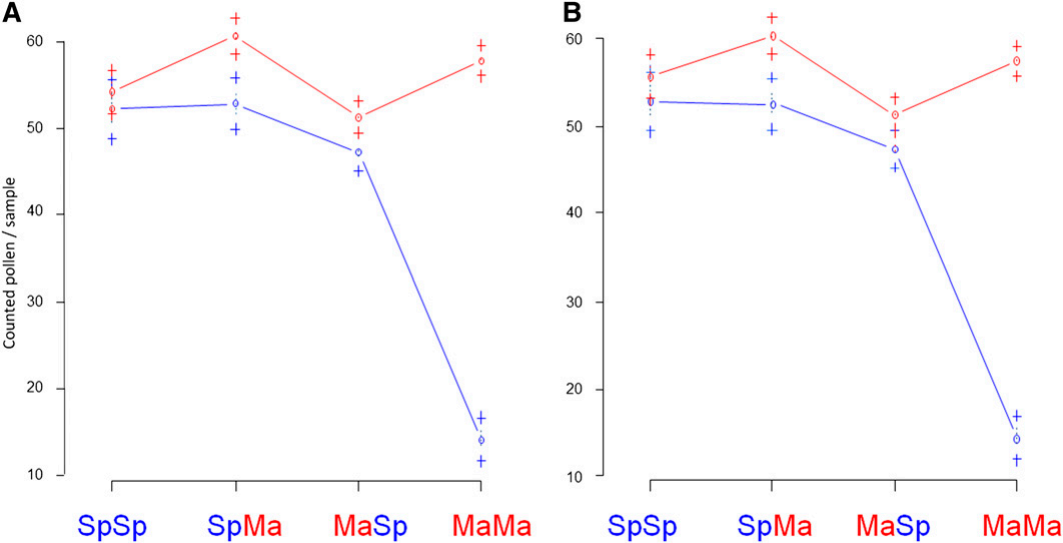
Effect plots of the closest markers of the pollen number quantitative trait loci (QTL) at the top of chromosome 2. The numbers of good pollen/sample produced by different genotypes at two markers, between which there is highest probability of QTL for cytoplasmic male sterility fertility-restorer gene. Blue represents Sp cytoplasm and red represents Ma cytoplasm. (A) AT1G63100. (B) AT1G62950.

The high marker density on chromosome 2 allowed us to fine-map the *Rf* locus to 2.00–4.35 cM, corresponding to 750–1345 kb (Figure 5). There was a striking difference in pollen production between the genotypes (Figure 6), with this QTL explaining 27% of the pollen number variation in SpMaF2. We detected no other cytoplasm-dependent QTL for pollen number but one independently acting QTL in chromosome 7 (Figure 5, Figure S5), explaining 3.2% of the total pollen number variation. For pollen viability, measured as the proportion of stained pollen grains in a sample, all the QTL found were cytoplasm-specific. For MaSpF2, we found four QTL [two with *P* < 0.001 (in chromosomes 2 and 4) and two with *P* < 0.05 (in chromosomes 7 and 8)]. The QTL in chromosome 2, explaining 2.3% of total pollen viability variation, had the highest LOD score of all pollen viability QTL. The highest probability for the location was at 3.61 cM, corresponding to ≈1180 kb, which is very close to the QTL for *Rf*. The low pollen viability caused by this locus was due to an interaction between the Ma cytoplasm and one of the two Sp alleles. When this Sp allele was present average pollen viability was 0.79, and when absent it was 0.84. For SpMaF2, we found only one QTL (*P* < 0.05) for pollen viability, which was located in chromosome 8 (Figure 5). We could not confirm any of the minor QTL related to pollen or seed production observed by Leppälä and Savolainen (2011).

### Transmission ratio distortion modifies expected phenotypic ratios

We compared only transmission of the Sp and Ma alleles in the whole data set without examining details of possible differences between the two alleles from the same population. In the combined F2 data, the range of transmission of Sp alleles across the genome varied from 0.39 to 0.6, as indicated in Figure 5. They were favored in large areas of chromosomes 1, 3, 7, and 8, whereas Ma alleles were more abundant in the first half of chromosome 2 and in the whole chromosome 4, except at the last marker, where there was an excess of SpMa heterozygotes instead. In addition, there were some single markers where Sp alleles were favored, and one marker in chromosome 6 where there was lack of SpMa heterozygotes.

In principle, we can predict phenotypic H:MS ratios of crossing progenies based on different inheritance models. Transmission ratio distortion, however, modifies these predictions. At the top of chromosome 2, where the restorer locus *Rf* is located (Leppälä and Savolainen 2011), the nonrestoring Ma alleles are transmitted more frequently than the restorer alleles of Sp. Instead of the expected 1:1 ratio, the alleles in the F2 were found with frequencies 0.575 and 0.425, respectively. If we assume a one-restorer locus model and take into account the TRD, then the expected number of MS SpMaF2 plants (homozygous for the Ma allele) would be 0.575^2^ * 694 ≈ 229, but we observed 144 instead, which is 62.8% of the expected number. Based on genotyping, we know that 40% of the Ma homozygotes (the plants having no *Rf* alleles) carrying the Sp cytoplasm are not MS. Thus, the Ma alleles at this locus are able to restore the fertility in 4 out of 10 cases (assuming there are no other loci affecting the trait). Other loci for *Rf* are extremely unlikely because we do not see any signs of such a QTL despite high power in our QTL analysis. When both transmission ratio distortion and 40% restoration of Ma alleles are taken into account, the one-locus model can well-explain the observed numbers of MS plants (Table 2). If we assume similar TRD in backcrosses, then we would expect 0.575 * 113 ≈ 65 MS [SpMa]Ma backcross plants, but we observed only 42, which is 64.6% of the expected. This suggests that the Ma alleles are able to restore the male fertility in 40% of the cases in these other crosses as well.

**Table 2:** Observed H:MS ratios of F2 and backcross hybrids compared to predictions by one-restorer locus model

H:MS			A	B
Cross	Observed	d.f.	Dominant	Dominant (40% Restoration by Recessive Allele)
F2	550:144	1	465:229*	556:138 (n.s.)
BC1	71:42	1	48:65*	74:39 (n.s.)
BC2 (H)	528:503	1	438:593*	547^a^:484 (n.s.)

F2 = SpMaF2, BC1 = [SpMa]Ma, BC2(H) = [SpMa]MaMa offspring of H [SpMa]Ma. A: Expected by one dominant locus. χ^2^ test of statistical significance compared with observed. B: Dominant restorer locus with 40% restoration of male sterility by the recessive allele. χ^2^ test of statistical significance compared with observed. H, hermaphrodite; MS, male-sterile; d.f., degrees of freedom; n.s., not significant; F_r_, frequency of restored among H; T_R_, transmission of restorer; r_(R)_, restoration of male sterility by recessive alleles among restored plants; r_(N)_, restoration of male sterility by recessive alleles among nonrestored plants; N, total number of plants.

* *P* < 0.001.

a H(exp) = H(exp progeny of restored) + H(exp progeny of non-restored) = [F_r_ × (T_R_ + (1-T_R_) × (r_(R)_)) + (1-F_r_) × (r_(N)_)] × N.

### Further backcrosses show variable CMS restoration

To examine whether a similar restoration level is found in the following generations with more homogenous genetic background, we continued backcross-like crosses further. [SpMa]MaMa second-generation backcross progenies were created by crossing MS and H [SpMa]Ma plants with two Ma pollen donors. We first confirmed that there was no difference in pollen production between the progenies of the two fathers (Wilcoxon *P* = 0.34) and that the proportion of MS in the progenies were also the same (608/1066 and 107/201), suggesting that the two pollen donor plants had similar genotypes.

An unexpectedly low number of H plants appeared in the progenies of MS [SpMa]Ma mothers. There were, on average, only 20% (n = 373) restored male-fertile plants among the progenies of MS BC1 mothers, whereas an average of 40% was expected. Between the 24 families, the H:MS ratios varied from 0:1 to 1:0 (Table S1). To calculate expected numbers of H and MS progeny of the H [SpMa]Ma mothers, we need to consider that there are unrestored plants among the H mothers, and after doing so we obtain a new expected H:MS ratio. The observed data fit this expectation well (Table 2). That leads to the conclusion that the 40% restoration by Ma alleles holds only for progenies of the H plants.

Due to unexpected results of the BC2 progeny, we grew the BC3 generation, which had a pattern quite similar to the BC2. The H [SpMa]MaMa plants produced 38% MS [SpMa]MaMaMa offspring of the total 467 that flowered (Table S2). Backcrossing of MS [SpMa]MaMa yielded 84% MS [SpMa]MaMaMa progeny (of 243 total flowering), thus only 16% were H, *i.e.*, restored. This is less than the 20% (n = 199) of restored in the progeny of the MS mothers in the previous generation (*P* = 0.048), thus likely indicating a possible effect of increased Ma genomic background. It can mean that MA alleles at *Rf* locus have better restoration capability together with SP genetic background, and accumulating MA alleles in backcrosses reduce the restoration of male sterility. Thus, we may expect to see differences between backcross lines started from H or MS BC1 plants. However, there were no difference in H:MS ratios between the progenies of MS [SpMa]MaMa mothers having either H or MS [SpMa]Ma grandmothers (*P* = 0.61; Table S2).

### Restoration capabilities of Ma alleles vary

Most of the Ma alleles we had in our crosses were able to restore fertility in approximately 40% of the cases. However, because we used several Ma plants, we also found some variation in the trait. To generate the BC3 generation, we used 11 different Ma pollen donors that may differ in their ability to restore male sterility of Sp cytoplasm, which gave us an opportunity to examine possible variation of *Rf* in the Ma population. There were no differences in H:MS ratios between the progenies of five Ma fathers and the MS BC2 mothers. In contrast, two of 11 fathers used in crosses with H BC2 mothers produced fewer MS progeny than the other nine that followed the expected ratio (Table S3). The result suggests that these deviating fathers (NC3A6-4 and NC3A17) that are full sisters either carry restorer alleles that restore fertility in more than 40% of the cases or may have additional restorer genes. One of them (NC3A6-4) was also used in crosses with MS mothers but showed no unexpected H:MS ratios of progeny in these crosses (Table S3).

### CMS is polymorphic in Spiterstulen, Norway

**Figure 7:**
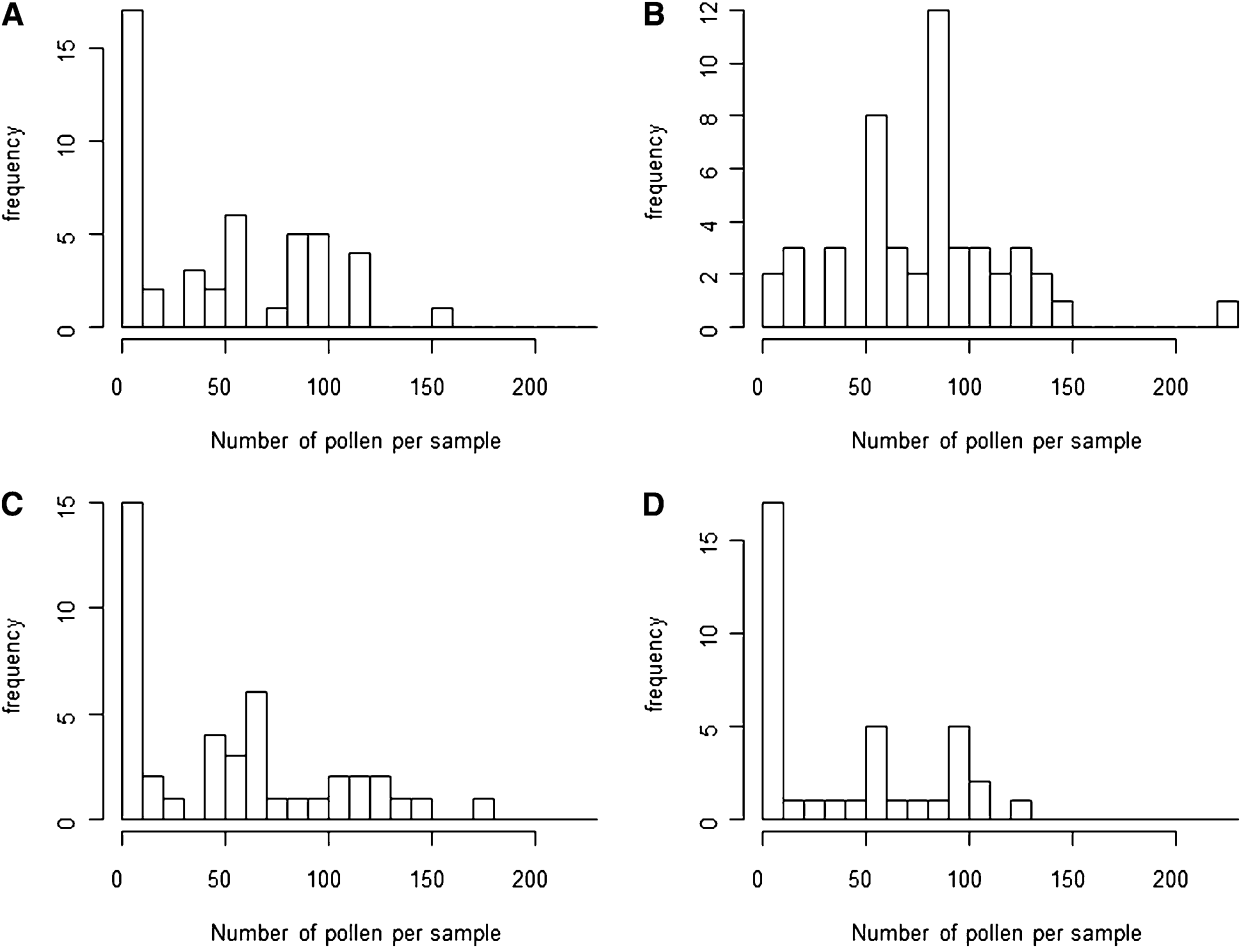
Family comparison. Number of pollen per sample for four (SpMa)Ma families in 2008 experiment. (A) Sp2. (B) Sp4. (C) Sp6. (D) Sp9. The same patterns are also seen among F2 families (data not shown).

To find possible polymorphisms in maternal CMS factor(s), we grew four half-sib families of both reciprocals of F2 and backcrosses. There were no differences between families with Ma cytoplasm, but the Sp4 (SpMa)Ma backcross family produced more pollen than backcross progeny of the other families with Sp cytoplasm (Kruskal–Wallis chi-squared = 18.8463; d.f. = 3; *P* = 0.0003; Figure S6). The F2 progeny of the SP4 family also seemed to produce more pollen, but with the low sample sizes this difference was not significant. The difference between family Sp4 and others was due to almost complete lack of male-sterile plants in family 4 (Figure 7). The Sp 4 family backcross plants also seem to produce better-quality pollen than the others (Figure S7), but the difference was not statistically significant. We conclude that the Sp4 cytoplasm does not carry CMS.

### No reduction in female fertility of the hybrids

Selection may favor MS plants (females) if they have better seed production than hermaphrodites. Thus, in the year 2007 experiment, we also examined seed production of the plants. The only significant differences (*P* < 0.05) in pairwise comparisons between different crosses and controls were the higher number of good seeds produced per silique by Ma control plants than produced by MaSpF1 and (SpMa)Ma hybrids. The latter also produced fewer good seeds than (MaSp)Ma plants (Figure S8). We did not find any QTL for seed fertility or any evidence of increased seed production of females, nor of tradeoffs between male and female reproductive fitness. Instead, there was a positive correlation between seed and pollen production in the SpMaF2 population, but not in (SpMa)Ma backcrosses (Figure S9). Plant rosette size and flowering shoot number after flowering were not correlated with pollen production in SpMaF2 (r = 0.050, *P* = 0.17 and r = 0.047, *P* = 0.20, respectively); however, in the (SpMa)Ma backcross plants, male fertility was positively correlated with plant rosette size (r = 0.313; *P* < 0.001) and negatively correlated with flowering shoot number (r = 0.221; *P* = 0.019).

## Discussion

### Mode of inheritance

The observed ratios of H and MS individuals in the F2 and BC1 progenies do not fit the one CMS and one recessive restorer locus model because there are too few MS compared to what would be expected. Based on this observation already seen by Leppälä and Savolainen (2011), they suggested that another restorer locus might be found. The first suggestion of our results, based on the sex ratios only, could be that the one dominant and one recessive restorer locus model can explain the observations. However, when information from genotyping markers close to the *Rf*, TRD, and advanced generation backcrosses are taken into account, the data are not consistent with a second locus. Instead, partial restoration by MA alleles of CMS in a one-locus model can account for the results, *i.e.*, many of the plants lacking restorers still express the H genotype. Thus, we conclude that there is a one CMS–one *Rf* system present in Sp.

In contrast to gynodioecious populations, in which balancing selection can maintain several polymorphic CMS-restorer systems (Dufay *et al.* 2007), we do not expect to find many fixed functional CMS-restorer systems when crossing diverged populations of hermaphrodite species. When both CMS and the corresponding restorers are fixed in natural populations, the selective advantage of the CMS gene disappears because it is not able to generate females any more. Then, only a possible cost of restoration (McCauley and Bailey 2009) remains, which favors mutations causing degeneration of the CMS gene in mitochondria and later inactivation of its restorers. New CMS can originate by rearrangements that create novel functional ORFs, when specific ORFs become associated with functional promoters. It is likely that the origin of new CMS is rarer than degeneration of existing ones because a new CMS mutation needs specific rearrangements, whereas any mutation may cause degeneration of existing ones. So, most hermaphrodite populations should lack functional CMS and restorers. Thus, cryptic CMS should come up only in some crosses and often only in one reciprocal direction, whereas crosses between other populations give fully hermaphroditic progeny.

Our data support this prediction. It seems that the fixation of CMS and corresponding *Rf* is not yet completed in Sp, whereas in Ma there are no functional CMS or restorers currently present. If the formation of new CMS genes is rarer than degeneration of existing ones, then the latter situation should be more common in natural populations. This seems to be the case at least in *Mimulus guttatus* and *M. nasutus*, where cytoplasm-dependent sterility has been found in 3 of 13 studied hermaphrodite populations (Martin and Willis 2010; Wise *et al.* 2011). Thus, these dynamics seem to be quite common at least in *Mimulus*, one of the best-studied plant genera. Further, in crosses between hermaphrodite lineages of crop species, as in rice (Li *et al.* 2007), a single locus has also been found. To our knowledge, there are no examples of multiple cryptic CMS-restorers systems existing in a single hermaphrodite wild population.

### Position of the *Rf* locus in the *A. lyrata* genome

Recent studies have shown that the class of PPR genes known to often be fertility restorers has evolved under positive selection. This provides evidence of an arms race between mitochondrial and nuclear genomes (Fujii *et al.* 2011). All of the members of this type of PPR gene in *A. lyrata* are located within a 4.4-Mb region in the chromosome 2 (628,493–5,039,588 bp) (Fujii *et al.* 2011). The point estimate for the location of the QTL we found for *Rf* is at 1,210,000 bp from the top of chromosome 2, thus within the region of the PPR genes. In the 95% Bayes interval of the QTL for *Rf* (750,000–1,345,000 bp), there are altogether 99 annotated genes in the *A. lyrata* reference genome. In that area, a Blast search reveals five complete and six partial PPR genes that are the strongest candidates for *Rf*.

Interestingly, we also found a pollen viability QTL at the same location as *Rf* in chromosome 2, but that was present only with Ma cytoplasm. This is an indication of a very weak CMS-like gene reducing pollen viability present in the Ma cytoplasm, which is restored by one of the PPR genes. Thus, at least in this case, these PPR genes may have a role in maintaining male reproductive function against cytoplasmic male sterility mutations.

Foxe and Wright (2009) found signs of diversifying selection at seven PPR loci they studied in *A. lyrata*. Two of these loci are located in chromosome 2, but not near the QTL detected in the present study. Our results pose the question whether the PPR genes within the QTL have evolved under positive selection, which would provide further evidence for arms race between mitochondrial and nuclear genomes. Further analyzing the sequences of these genes would lead us closer to identifying and cloning the restorer gene of the CMS found in Sp.

### Genotyping would help solve labile expression of CMS

The partial restoration of CMS by Ma alleles depended on the genomic background in our crosses. The MS BC2 (SpMa)MaMa plants with more Ma alleles in their genome compared to the BC1 produced fewer H progeny than MS BC1 plants. This is consistent with possible labile expression of CMS and/or complex inheritance of restorer genes, which have been observed, for instance, in *Plantago coronopus* (Koelewijn and van Damme 1995b; Koelewijn 2003), *Thymus vulgaris* (Belhassen *et al.* 1991), *Beta vulgaris* (Dufay *et al.* 2008), and *Silene vulgaris* (Charlesworth and Laporte 1998). Studying such cases further by genotyping the restorer loci would shed new light on CMS restoration systems in which the explicit genetic models have not fully accounted for the H:MS ratios. Progeny testing and measuring CMS of perennial plants during several growing seasons would also help provide more reliable estimates of individual plants’ genotypes.

### Influence of TRD-to-genotype ratios

The genetic analysis was influenced by the TRD present in crosses between these subspecies, and the genotyping was a necessary component for taking this into account both for examining the number of loci and the issues of partial restoration. In general, if a significant amount of TRD is present, then crossing ratios without any other genetic knowledge may lead to erroneous conclusions. The largest effects of TRD are more likely when crosses are between distantly related populations (Leppälä *et al.* 2013).

Surprisingly, the allele unable to restore CMS was favored as a part of large block in chromosome 2 with more Ma alleles than expected. The highest TRD maps approximately 2 cM downstream of the *Rf* locus indicate that the loci are different. The reason for TRD is unknown, but nothing suggests it has something to do with the CMS. As recent results by Leppälä *et al.* (2013) indicate, the severe TRD linked to *Rf* observed here is most likely not present in more closely related populations unless there are, for instance, meiotic drive genes present and closely linked to restorer loci (Leppälä and Savolainen 2011; Phadnis and Orr 2009).

### Male and female fertility

If the seed production of female plants is increased, then selection favors the spread of CMS in the population. Otherwise, CMS must have spread due to genetic drift, and there is no potential for stable CMS polymorphisms (Charlesworth 1981; Dufay *et al.* 2007). We did not find such an effect by comparing pollen and seed production per flower. This is not surprising because per-flower seed production of female plants is seldom increased in gynodioecious populations, whereas total seed production is more often increased (Shykoff *et al.* 2003). In (SpMa)Ma backcross plants, we observed that pollen production was correlated negatively with flower shoot number and positively correlated with leaf rosette size. However, the cause of this is not the CMS, but a strong QTL for flower shoot number linked to *Rf* locus (our own unpublished QTL mapping results of the current cross and personal communication with David Remington 2012). Because of this kind of different resource allocation, measuring female fitness precisely would require estimating total seed production over multiple years. To conclude, we did not observe any signs of increased seed production of females compared with hermaphrodites in a single-year experiment.

For these two *A. lyrata* populations, the reduction of male fertility in the hybrids has taken place during evolution before any significant female effects, as was found by Leppälä and Savolainen (2011). In monkey flowers, both pollen and seed fertility of *M. guttatus* × *M. nasutus* hybrids were decreased (Fishman and Willis 2001); in tomato *Lycopersicon hirsututm* × *L. esculentum* hybrids, there is even more reduction in seed production than in pollen viability (Moyle and Graham 2005). In many animals such as *Drosophila* flies, in contrast, hybrid male sterility and inviability usually evolve first, mainly due to the effects of sex chromosomes (Presgraves 2008), but other reasons are also possible.

### Within-population polymorphism of CMS and *Rf*

There are two ways to explain the difference observed in reciprocal crosses. The most likely explanation is that there is no CMS present in the Ma population, thus the plants having Ma cytoplasm are always hermaphrodites. Plants of the Ma population also lack complete restorers for the CMS of Sp, but there seem to be rare partial restorers, because some Ma plants crossed to CMS cytoplasm containing pollen recipients have lower than expected numbers of MS plants in their progeny. This indicates that there may have been functional CMS also in the history of Ma, after which some restorers are still present. The other, less parsimonious explanation for the reciprocal difference is that there are CMS and restorers present also in Ma. Then, the plants of Sp should have restorers that are able to restore male fertility of both Sp and Ma cytoplasms, whereas Ma restorers are not able to restore CMS of Sp. Crosses involving more populations are needed to solve this issue.

There is cytoplasmic variation in Sp, because one of the four cytoplasms tested failed to segregate male sterility. Field observations indicate that there are also some plants lacking restorers in Sp, because rare MS plants are found in the wild (Päivi Leinonen and Johanna Leppälä, 2012 personal communication). Thus, it seems that the fixation process of CMS and restorers are still ongoing in this population, a condition that, to our knowledge, has not been reported in nature so far.

### Symmetric incompatibilities due to NMS or symmetrical CMS

Symmetric reproductive barriers are not necessarily attributable to nuclear genes but can be reciprocally similarly acting cytonuclear interactions. The observed reduced pollen viability of F1 and F2 hybrids was symmetric; however, in backcrosses, pollen viability (in addition to CMS) was dependent on the cytoplasm of the plant. Thus, it is evident that there is a cytoplasmic component in this trait, but based on phenotypic data only we cannot exclude possible interaction between nuclear loci.

The mapping of pollen viability in F2 revealed QTL at completely different loci in reciprocal SpMaF2 and MaSpF2 plants, which supports the hypothesis of nuclear cytoplasmic interaction. The strongest QTL for pollen viability was at the top of chromosome 2, almost at the same location as the QTL for *Rf*, but present only in one of the reciprocals, namely the F2s with Ma cytoplasm. The negative interaction was between Ma cytoplasm and one of the two Sp alleles. One explanation for this observation is that Ma cytoplasm has a gene or genes disrupting pollen development when interacting with the specific Sp allele. Alternatively, it is possible that partial male sterility caused by Ma cytoplasm is better-restored if two restorers are present and one of the two Sp alleles has less potential for restoration than the other Sp allele and both Ma alleles.

### CMS and speciation

Speciation in plants is unlikely to be a consequence of a single gene or isolating barrier (Lowry *et al.* 2008; Widmer *et al.* 2009). Postzygotic BDM incompatibilities are not regarded as a major cause of speciation in plants, because they typically correlate poorly with species boundaries and make small contributions to total isolation at later stages (Rieseberg and Willis 2007). How much could the incompatibilities presented here contribute to speciation of the subspecies of *A. lyrata* in case of possible secondary contact after isolation of 140,000 generations is unclear. The question is also largely theoretical because of large habitat and life history differences between the populations would likely cause of strong premating isolation. However, Leppälä and Savolainen (2011) did point to the role of cytonuclear interactions in the development of reproductive isolation between these populations.

In case of random mating at a hybrid zone, the male sterility observed here would arise in 60% of those F2s and backcrosses that have CMS cytoplasm of Sp and lack corresponding restorers, in other words, approximately 7.5% of all F2 and backcross hybrids. In addition, pollen viability and pollen production were reduced in hybrids that were not completely male-sterile. Altogether, production of good pollen grains per flower in the hybrids was approximately 80% of that of the average of the parental populations. Thus, the average male reproductive fitness of the hybrids is three-fourths compared with parental populations. However, other fitness components may have different patterns. For instance, hybrids had higher survival and seed production than parents when grown in the wild in Norway, but not in North Carolina (Leinonen *et al.* 2011). Thus, the overall hybrid fitness likely depends on the environment.

More importantly, CMS and restorers arise and disappear cyclically and spread rapidly through hermaphroditic populations. Hence, when hermaphrodite populations with different CMS and dominant restorers come into secondary contact, it would most likely lead to fixation of a functional CMS-restorer system throughout the hybrid population, just as it does in populations in which a new CMS mutation arises (Charlesworth and Ganders 1979). As Fishman and Willis (2006) suggested, the introgression of both CMS and its restorer through the whole hybrid population may be even faster because of their high initial frequency. Thus, the case is very different compared with those reproductive barriers that cause a permanent reduction in fitness of hybrids between diverged populations.

Fishman and Willis (2006) discussed further the dynamics of CMS in hybrid zones and its contribution as a postzygotic isolating barrier. Although a cryptic CMS in *Mimulus* was observed between an outcrossing and a selfing species with no self-incompatibility system, Fishman and Willis (2006) suggest that it may also be a common source of both symmetric and asymmetric isolating barriers in a secondary contact of previously allopatric outcrossing populations. Here, we present some additional thoughts based on the present data of two populations of outcrossing species with strong self-incompatibility system.

It is clear that both CMS and the corresponding restorer will go to rapid fixation if there is increased seed production in females, but there is no potential for stable polymorphism of females and hermaphrodites (Charlesworth and Ganders 1979; Hanson and Bentolila 2004). Stable polymorphism is even more unlikely in self-incompatible species, in which there is no benefit from the ability of the females to avoid selfing. However, even without any selective advantage, CMS may spread via drift, because loss of male reproductive fitness has no effect on the spread of the maternally inherited cytoplasmic genome. In this case, selection favoring fixation of corresponding restorer should be even stronger.

CMS may be evolving mainly at the local scale, as has been observed in *Mimulus* (Fishman and Willis 2006; Martin and Willis 2007; Martin and Willis 2010) and, as would be generally expected, because of spread of CMS via seeds only. The appearance of CMS in between subspecies crosses will thus depend on the populations considered. As discussed, most hermaphrodite populations should lack functional CMS and restorers, making asymmetric hybrid male sterility more likely, thus keeping CMS dynamics in hybrid zones simpler. In the current case, and different from *Mimulus*, where the nonrestored hybrids were completely male-sterile (Fishman and Willis 2006), the restorers not matching the CMS were still able to restore male fertility of 40% of the hybrids. This has no effect on the spread of maternally inherited CMS but allows more introgression of nuclear genomes between populations, and thus speeds up formation of a stable hybrid population. To conclude, CMS would not permanently prevent gene flow in case of secondary contact of the two *A. lyrata* populations studied, and we hypothesize that dynamics of CMS may be limited in initiating speciation of allopatric outcrossing populations. However, because cryptic CMS seems to be quite common in *Mimulus* and now has also been observed in *Arabidopsis*, it clearly has potential as an interesting part of metapopulation dynamics (Pannell and Charlesworth 2000) in many plant species.

## Supplementary Material

Supporting Information
