# Supplementary material for: Cytoplasmic Male Sterility Contributes to Hybrid Incompatibility Between Subspecies of Arabidopsis lyrata
Source: G3 (Bethesda). 2013 Oct 1;3(10):1727–40. doi: 10.1534/g3.113.007815 (PMC3789797; doi:10.1534/g3.113.007815)
Supplement: Supporting Information [file supp_3_10_1727__index.html]

Cytoplasmic Male Sterility Contributes to Hybrid Incompatibility Between Subspecies of Arabidopsis lyrata — Supporting Information 

# Cytoplasmic Male Sterility Contributes to Hybrid Incompatibility Between Subspecies of *Arabidopsis lyrata*

## Supporting Information for Aalto, Koelewijn, and Savolainen, 2013

**Files in this Data Supplement:**

- Supporting Information - Figures S1-S9, Files S1-S4, and Tables S1-S3 (PDF, 1 MB)
- Figure S1 - Crossing scheme (PDF, 248 KB)
- Figure S2 - Pollen numbers per sample of the plants in the greenhouse experiments ranked by increasing order (PDF, 206 KB)
- Figure S3 - Pollen viability summary of 2007 experiment (PDF, 310 KB)
- Figure S4 - Pollen production of good and bad anthers (PDF, 307 KB)
- Figure S5 - Two QTL interaction scan for *rf* in SpMaF2 (PDF, 420 KB)
- Figure S6 - Pollen number summary of 2008 experiment (PDF, 195 KB)
- Figure S7 - Pollen viability summary of 2008 experiment (PDF, 287 KB)
- Figure S8 - Seed count (PDF, 294 KB)
- Figure S9 - Correlation of seed and pollen production (PDF, 213 KB)
- Table S1 - H:MS ratios of BC2 families (PDF, 238 KB)
- Table S2 - H:MS ratios of BC3 (PDF, 214 KB)
- Table S3 - Sex ratios of BC3 progenies by mother's sex and father (PDF, 306 KB)
- File S1 - Genotypes (.xlsx, 990 KB)
- File S2 - Phenotypes 2007 (.xls, 668 KB)
- File S3 - Phenotypes 2008 (.xls, 499 KB)
- File S4 - Phenotypes 2009 (.xls, 125 KB)
