# Supplementary material for: Cytoplasmic Male Sterility Contributes to Hybrid Incompatibility Between Subspecies of Arabidopsis lyrata
Source: G3 (Bethesda). 2013 Oct 1;3(10):1727–40. doi: 10.1534/g3.113.007815 (PMC3789797; doi:10.1534/g3.113.007815)
Supplement: Supporting Information [file supp_g3.113.007815_007815SI.pdf]

G3 Genes | Genomes | Genetics

Cytoplasmic Male Sterility contributes to hybrid incompatibility between subspecies of *Arabidopsis lyrata*

Esa A. Aalto*, Hans-Peter Koelewijn§, Outi Savolainen†

* Department of Biology, and †Biocenter Oulu, University of Oulu, Oulu, Finland, FIN-90014
§ Nunhems Netherlands B.V., Haelen, The Netherlands

DOI: 10.1534/g3.113.007815

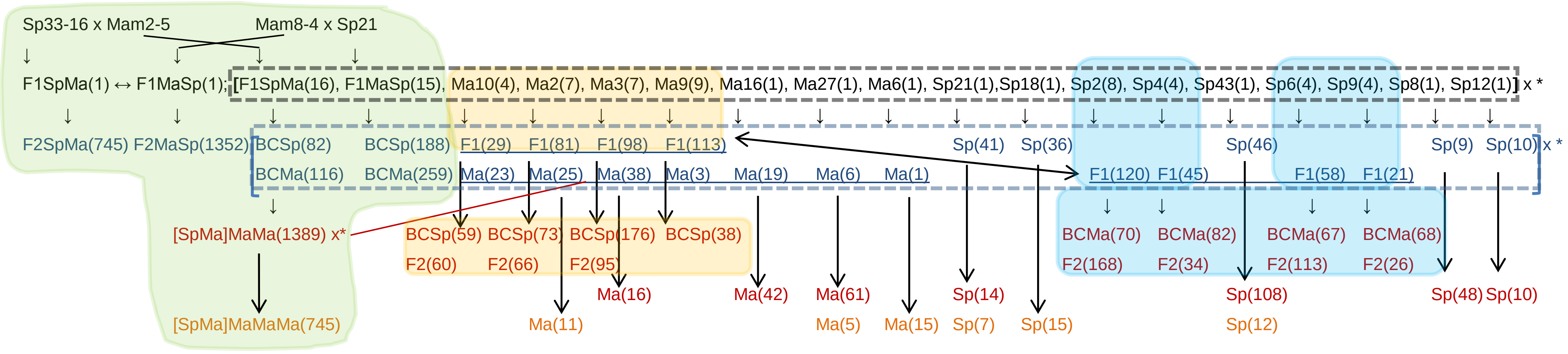

* 2 pollinator plants: Sp38-23, Ma7-10

* 4 pollinator plants: Sp19, Sp11-3, Ma22-1, Ma29-2

* 11 pollinator plants: Ma10-6/13, Ma10-6/15, Ma2-8/10, Ma2-8/16, Ma3A/6-4, Ma3A/6-5, Ma3A/12, Ma3A/13, Ma3A/17, Ma3A/18, Ma16-6x12-2/5

Figure S1 Crossing scheme. Years are indicated by colors: 2005 (or earlier), 2007, 2008, 2009. x = one way cross. ↔ = two way cross. Arrows indicate progeny from the mother plant. Numbers in parentheses indicate number of sister plants grown. Plants surrounded by brackets and dashed boxes are included in same crosses, pollinators of which are below the scheme. Light background colors indicate plants belonging to the same experiment: Green = main crossing family (F2s used in QTL mapping); yellow = four families for studying CMS polymorphism in Ma; blue = four families for studying CMS polymorphism in Sp. BCSp = backcross to Spiterstulen ([MaSp]Sp and [SpMa]Sp), BCMa = backcross to Mayodan ([MaSp]Ma and [SpMa]Ma).

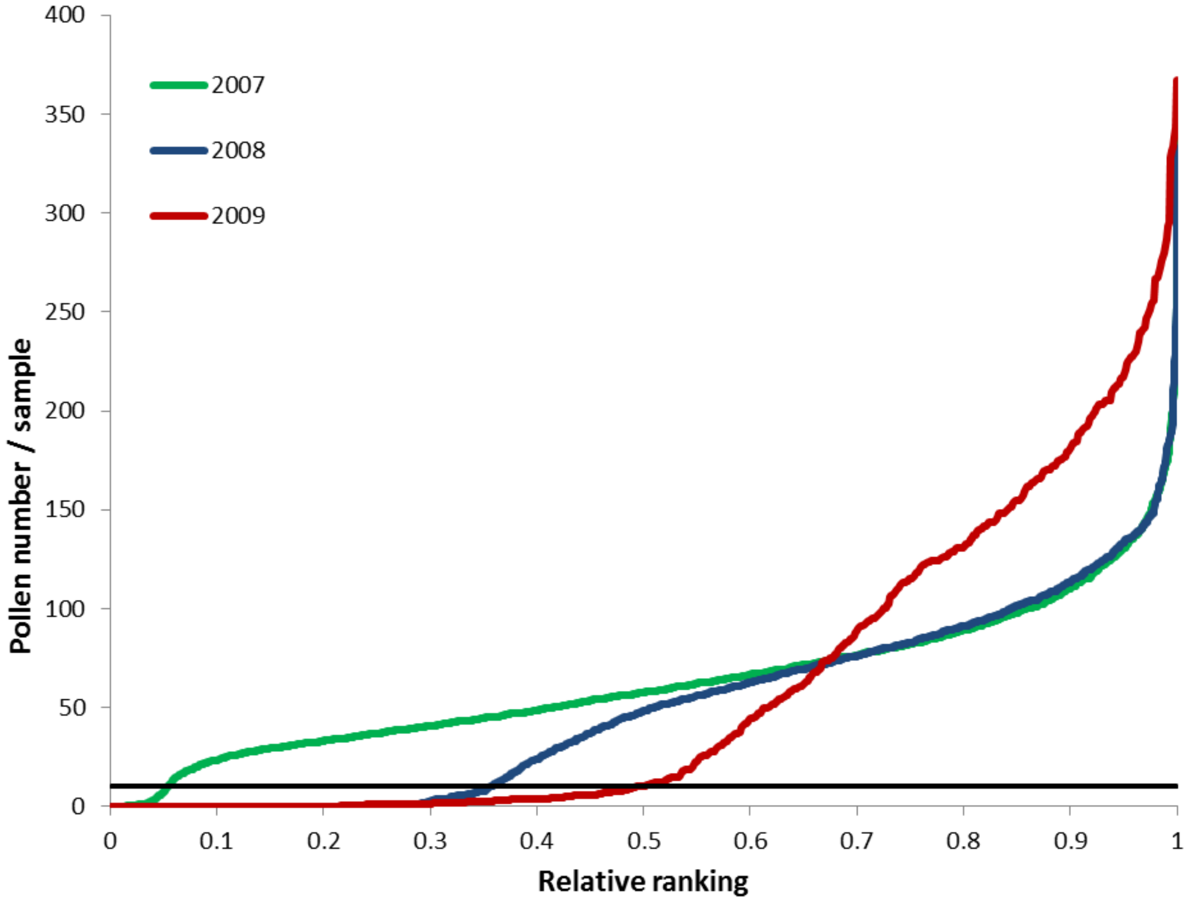

Figure S2 Pollen numbers per sample of the plants in the greenhouse experiments ranked by increasing order. Black vertical line indicates threshold value below which the plants were classified as male sterile.

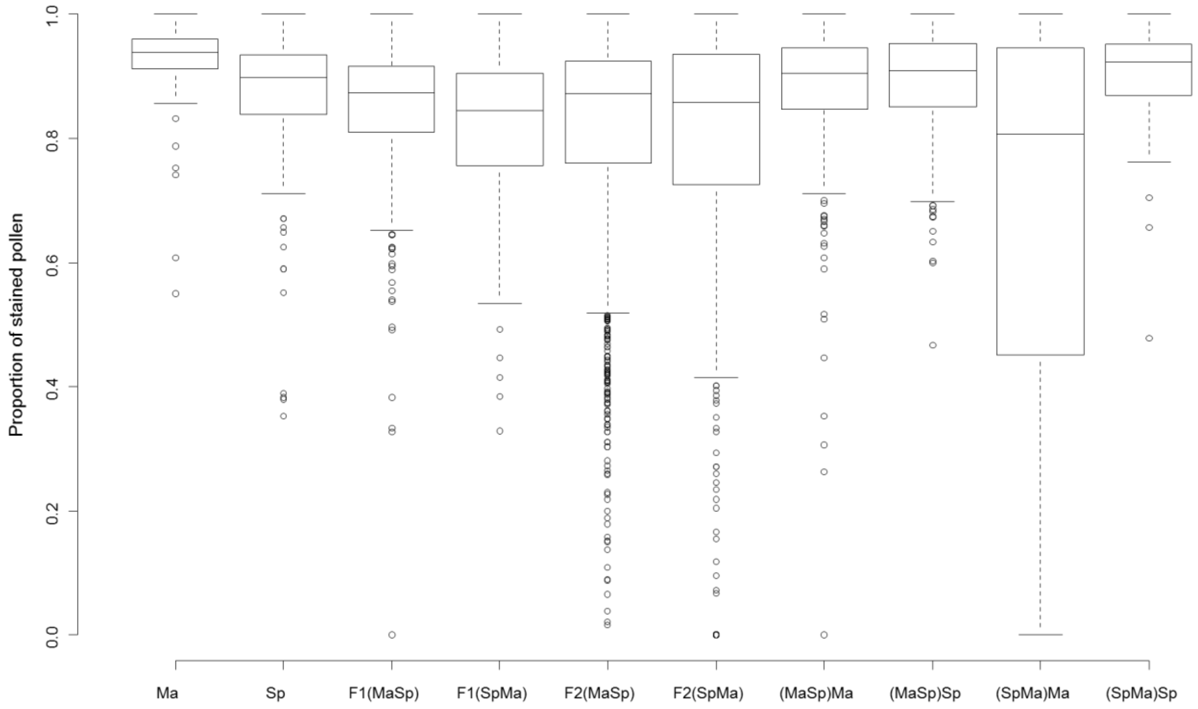

Figure S3 Pollen viability summary of 2007 experiment. Proportion of good (stained) pollen (horizontal line: median, box: quartiles, dots: outliers) for parental populations, F1 and F2 hybrid reciprocals and four types of backcrosses.

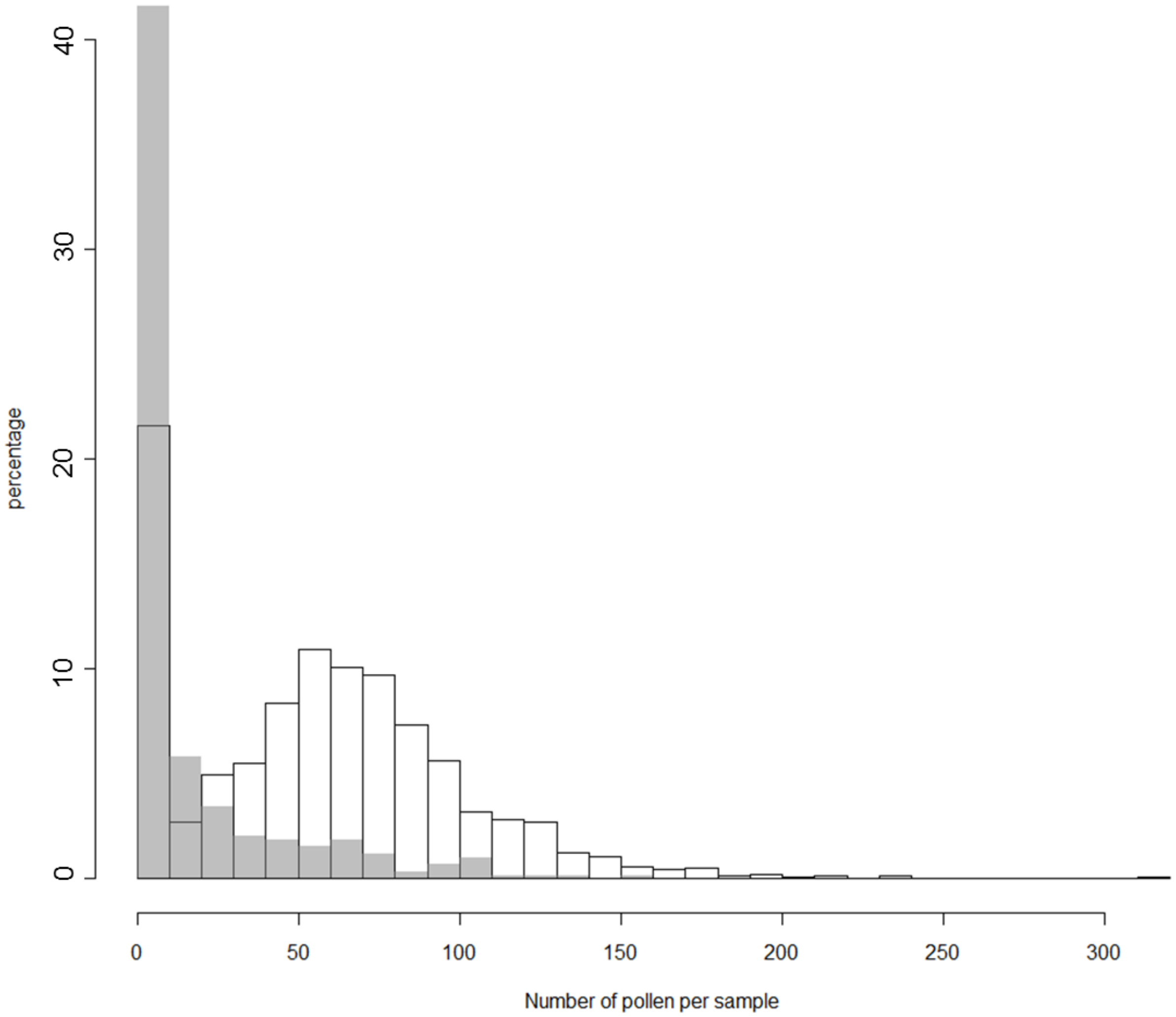

Figure S4 Pollen production of good and bad anthers. Number of pollen per sample produced by plants with poor anthers (grey shading) and plants having normally looking anthers (black borders) in year 2008 experiment.

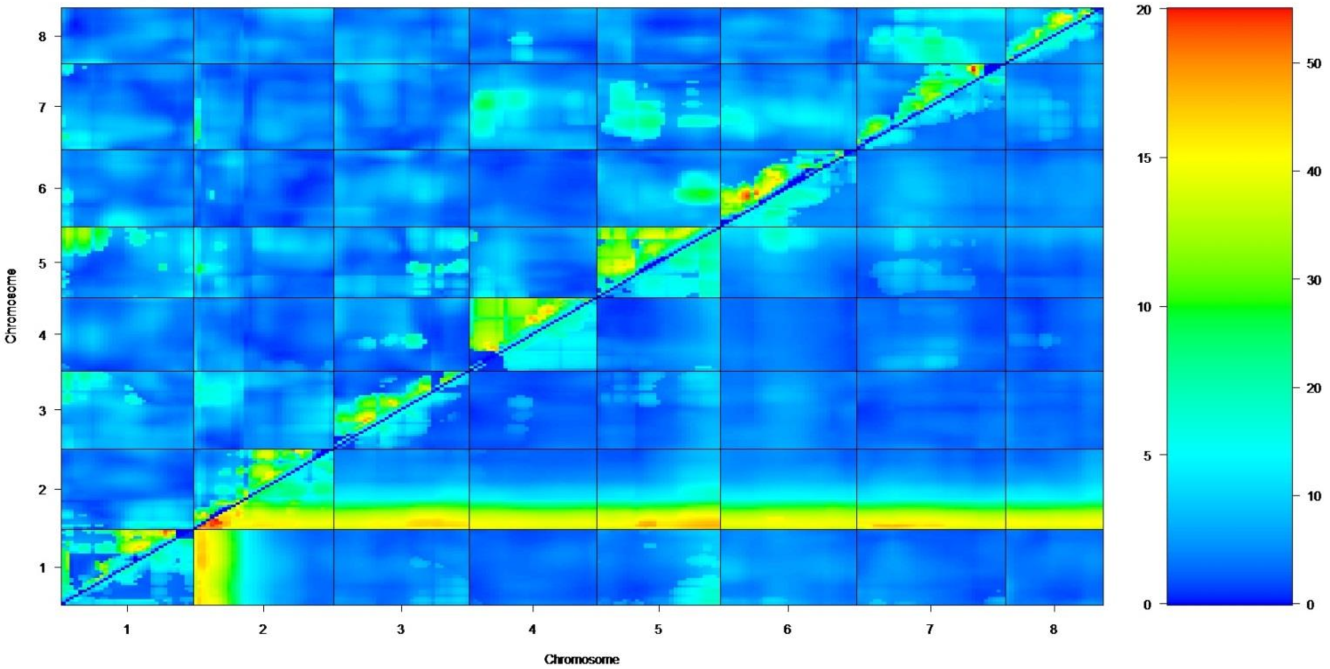

Figure S5 Two QTL interaction scan for *rf* in SpMaF2. The QTL at the beginning of chromosome 2 is clearly visible, but there are not any interactions with it and other loci.

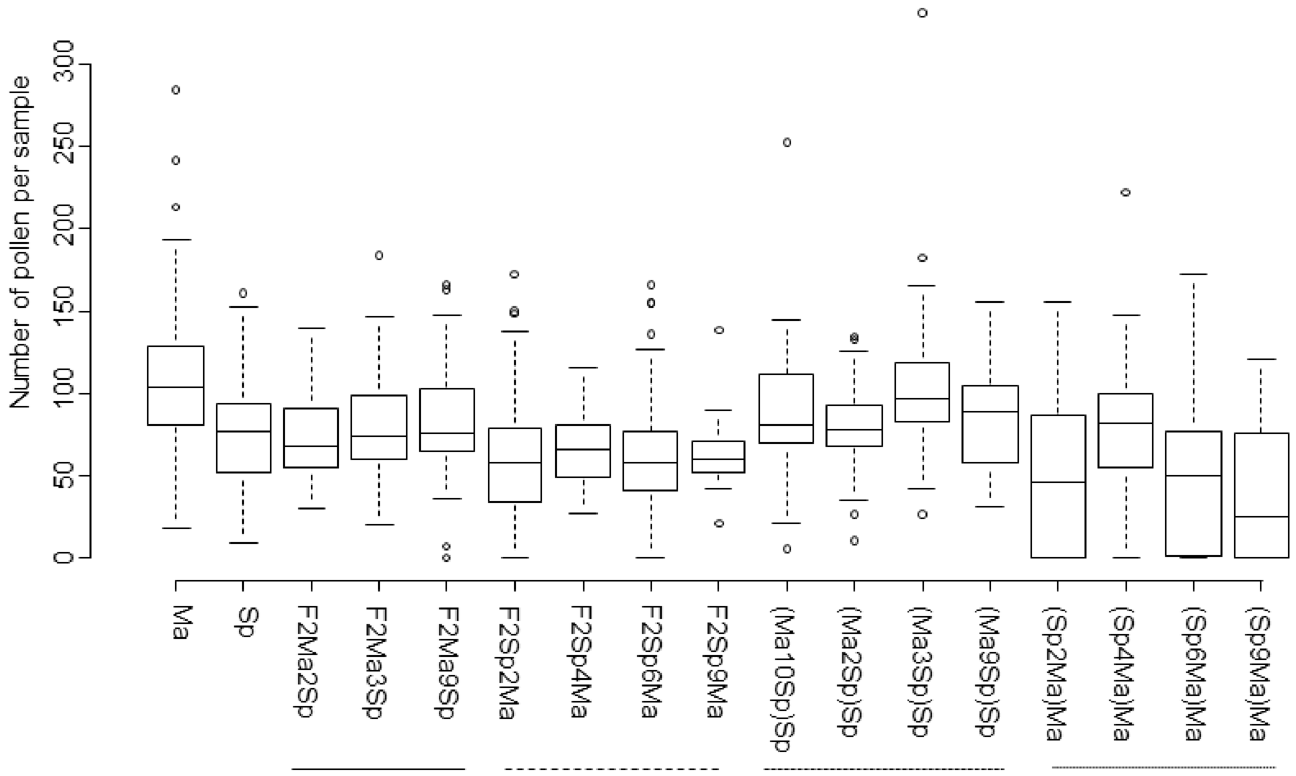

Figure S6 Pollen number summary of 2008 experiment. Number of pollen per sample (horizontal line: median, box: quartiles, dots: outliers) for parental populations, 3 MaSpF2 families (continuous line), 4 SpMaF2 families (dashed line), 4 (MaSp)Sp families (square-dotted line) and 4 (SpMa)Ma families (round-dotted line).

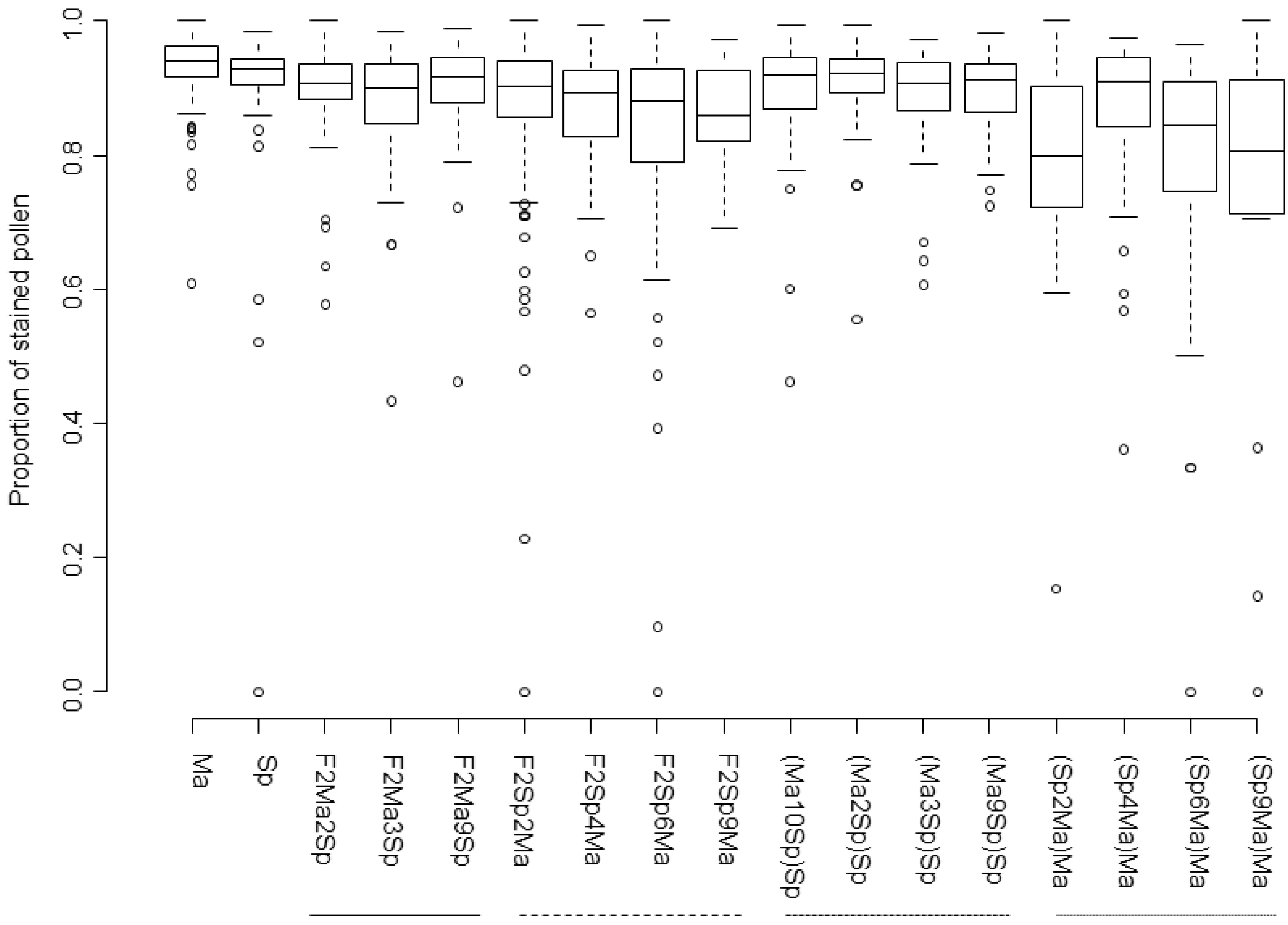

Figure S7 Pollen viability summary of 2008 experiment. Proportion of good (stained) pollen (horizontal line: median, box: quartiles, dots: outliers) for parental populations, 3 MaSpF2 families (continuous line), 4 SpMaF2 families (dashed line), 4 (MaSp)Sp families (square-dotted line) and 4 (SpMa)Ma families (round-dotted line).

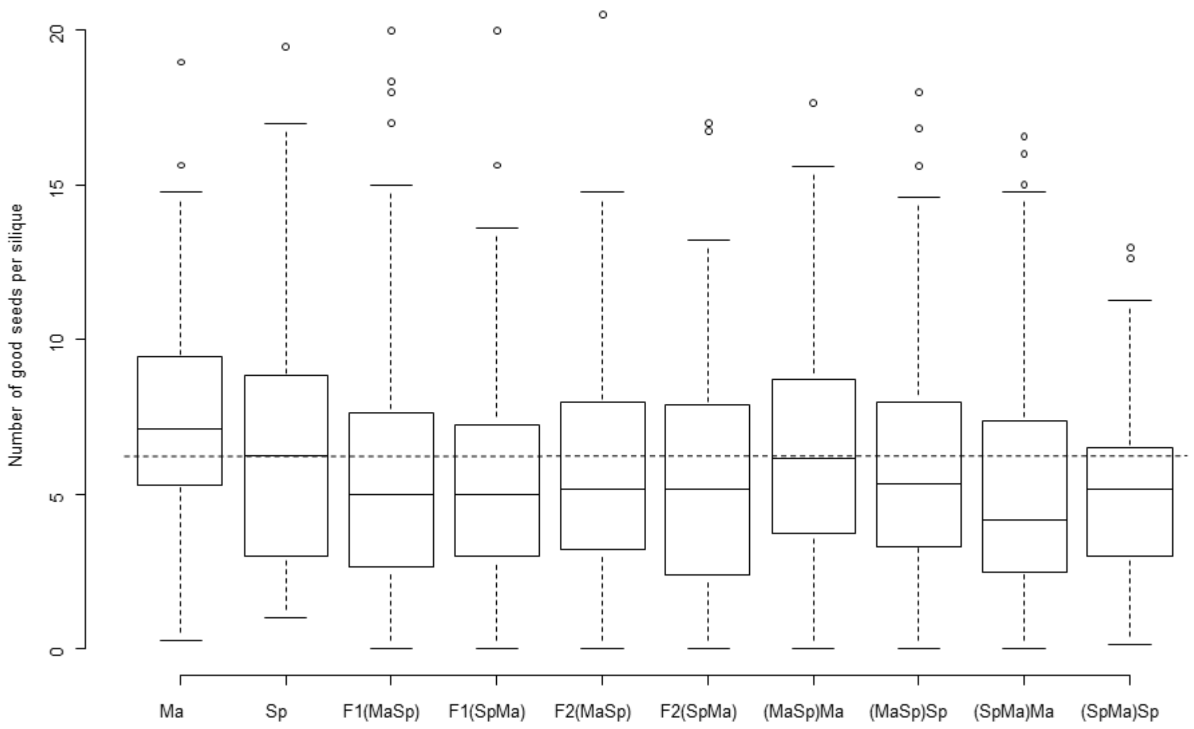

Figure S8 Seed count. Number of good seeds per sample produced by plants in year 2007 experiment (horizontal line: median, box: quartiles, dots: outliers) for parental populations, F1 and F2 hybrid reciprocals and four types of backcrosses. Dashed horizontal line indicates lower parental (Sp) mean seed production. None of the pairwise differences between Sp and the others were significant in Kruskall-Wallis test (total p = 0.00045).

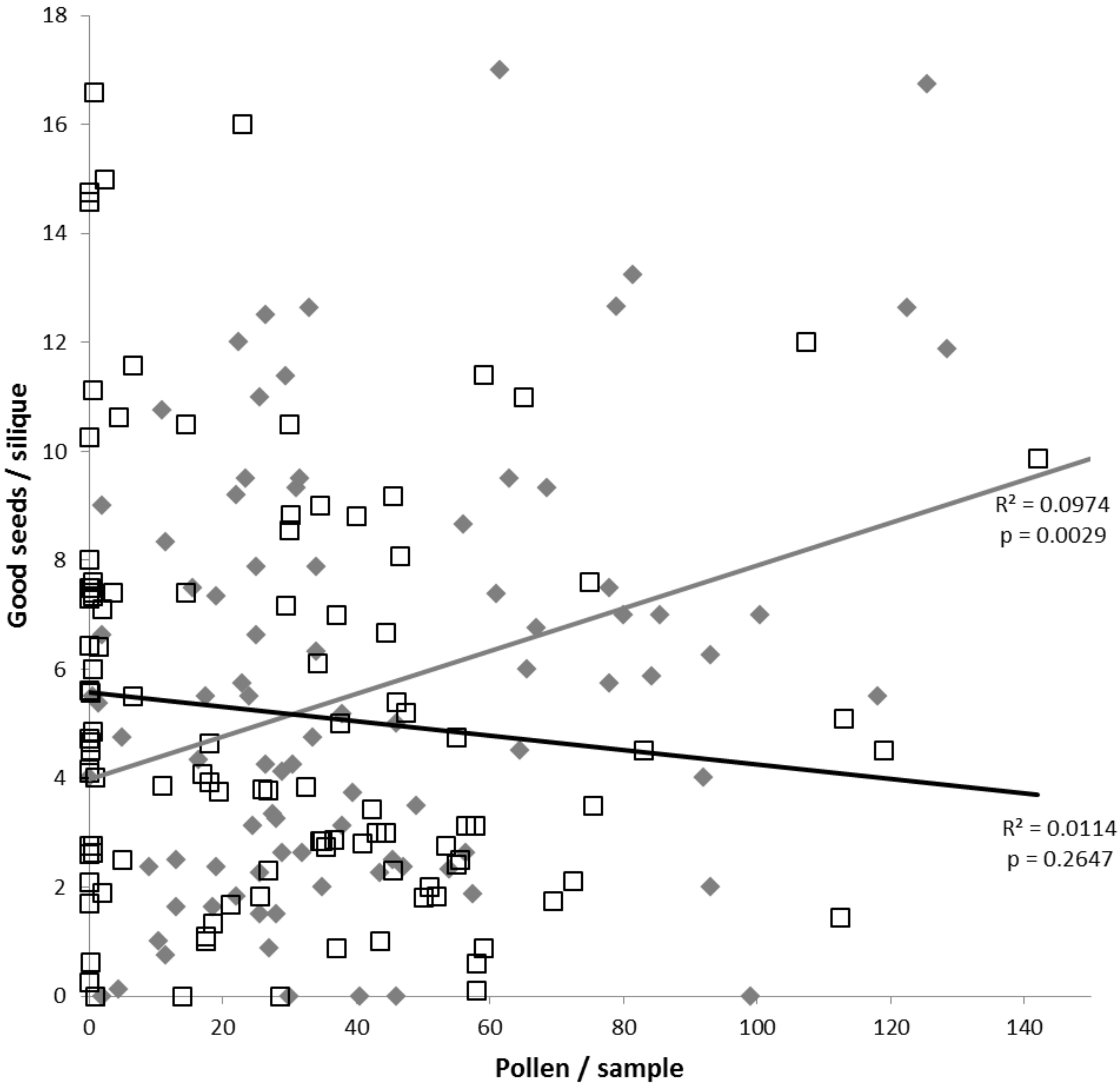

Figure S9 Correlation of seed and pollen production. Male and female fertility of (SpMa)F2 (grey diamonds) are positively correlated, but in (SpMa)Ma backcross plants (open squares) there is no significant correlation.

Files S1-S4

Available for download at http://www.g3journal.org/lookup/suppl/doi:10.1534/g3.113.007815/-/DC1

File S1 Genotypes

File S2 Phenotypes 2007

File S3 Phenotypes 2008

File S4 Phenotypes 2009

Table S1 H:MS ratios of BC2 families. Almost all MS BC1 mothers raised mostly MS progeny, but there was more variation in H:MS ratios of H mothers.

Mother	Father	Sex mother	H	MS
SpMaMa14-10	NC22-1	H	5	7
SpMaMa14-4	NC22-1	H	2	1
SpMaMa14-5	NC22-1	H	3	13
SpMaMa14-6	NC22-1	H	8	9
SpMaMa15-1	NC22-1	H	3	7
SpMaMa15-16	NC22-1	H	4	12
SpMaMa15-17	NC22-1	H	3	0
SpMaMa15-8	NC22-1	H	2	0
SpMaMa15-9	NC22-1	H	3	13
SpMaMa1-10	NC22-1	H	32	13
SpMaMa1-16	NC22-1	H	12	5
SpMaMa1-2	NC22-1	H	1	12
SpMaMa1-3	NC22-1	H	2	6
SpMaMa1-5	NC22-1	H	7	6
SpMaMa1-6	NC22-1	H	5	32
SpMaMa1-7	NC22-1	H	4	4
SpMaMa8-11	NC22-1	H	5	0
SpMaMa8-2	NC22-1	H	1	1
SpMaMa8-3	NC22-1	H	9	6
SpMaMa8-7	NC22-1	H	2	2
SpMaMa8-8	NC22-1	H	9	5
SpMaMa8-9	NC22-1	H	5	0

SpMaMa10-1	NC22-1	H	4	11
SpMaMa10-8	NC22-1	H	0	3
SpMaMa11-11	NC22-1	H	3	0
SpMaMa11-14	NC22-1	H	6	1
SpMaMa11-15	NC22-1	H	11	1
SpMaMa11-17	NC22-1	H	4	1
SpMaMa11-18	NC22-1	H	17	0
SpMaMa11-19	NC22-1	H	0	2
SpMaMa11-21	NC22-1	H	12	2
SpMaMa11-4	NC22-1	H	7	2
SpMaMa11-6	NC22-1	H	3	0
SpMaMa11-7	NC22-1	H	3	2
SpMaMa11-8	NC22-1	H	14	0
SpMaMa11-9	NC22-1	H	9	2
SpMaMa16-11	NC22-1	H	2	2
SpMaMa16-2	NC22-1	H	19	7
SpMaMa16-3	NC22-1	H	3	8
SpMaMa16-4	NC22-1	H	12	22
SpMaMa16-5	NC22-1	H	1	2
SpMaMa16-6	NC22-1	H	6	2
SpMaMa16-7	NC22-1	H	21	10
SpMaMa16-7	NC22-1	H	21	10
SpMaMa16-8	NC22-1	H	3	7
SpMaMa9-11	NC22-1	H	26	16
SpMaMa9-17	NC22-1	H	10	10

SpMaMa9-19	NC22-1	H	22	15
SpMaMa9-21	NC22-1	H	10	12
SpMaMa9-22	NC22-1	H	3	2
SpMaMa9-23	NC22-1	H	7	20
SpMaMa9-3	NC22-1	H	14	54
SpMaMa9-4	NC22-1	H	1	1
SpMaMa9-7	NC22-1	H	4	29
SpMaMa9-8	NC22-1	H	23	12
SpMaMa14-5	NC29-2	H	5	4
SpMaMa15-10	NC29-2	H	62	79
SpMaMa1-1	NC29-2	H	6	2
SpMaMa1-12	NC29-2	H	1	1
SpMaMa1-7	NC29-2	H	5	1
SpMaMa11-12	NC29-2	H	4	0
SpMaMa11-7	NC29-2	H	8	6
SpMaMa14-1	NC22-1	MS	1	8
SpMaMa14-2	NC22-1	MS	0	8
SpMaMa14-8	NC22-1	MS	1	28
SpMaMa15-11	NC22-1	MS	0	1
SpMaMa15-13	NC22-1	MS	2	4
SpMaMa15-14	NC22-1	MS	2	3
SpMaMa15-7	NC22-1	MS	0	5
SpMaMa1-13	NC22-1	MS	1	14
SpMaMa8-1	NC22-1	MS	2	5
SpMaMa8-12	NC22-1	MS	1	1

SpMaMa10-5	NC22-1	MS	3	11
SpMaMa10-6	NC22-1	MS	9	0
SpMaMa10-7	NC22-1	MS	0	3
SpMaMa11-10	NC22-1	MS	3	4
SpMaMa11-2	NC22-1	MS	9	9
SpMaMa9-1	NC22-1	MS	4	9
SpMaMa9-13	NC22-1	MS	0	11
SpMaMa9-15	NC22-1	MS	2	7
SpMaMa9-16	NC22-1	MS	1	11
SpMaMa9-18	NC22-1	MS	1	19
SpMaMa9-20	NC22-1	MS	1	2
SpMaMa9-6	NC22-1	MS	2	11
SpMaMa9-9	NC22-1	MS	3	11
SpMaMa14-2	NC29-2	MS	0	3
SpMaMa15-18	NC29-2	MS	1	6
SpMaMa15-5	NC29-2	MS	1	5

Table S2 H:MS ratios of BC3. Fathers NC3A/6-4 and 4NC3A/17 produced excess of H progeny (see table S3).

Father	Mother	Sex mother	Sex grandmother	H	MS
NC3A/6-4	1265	H	H	13	5
NC3A/6-4	1400	H	MS	16	5
NC3A/6-4	1957	H	H	17	6
NC3A/17	51	H	H	18	2
NC3A/17	93	H	H	14	0
NC3A/17	393	H	H	10	1
NC10-6/13	22	H	H	11	8
NC10-6/13	289	MS	MS	2	10
NC10-6/13	476	MS	MS	7	22
NC10-6/13	2077	MS	MS	3	21
NC10-6/15	404	H	H	11	12
NC12, NC3A/12	110	H	H	15	1
NC16-6x12-2/5	2462	H	H	14	10
NC16-6x12-2/5	2925	H	H	8	9
NC3A/6-5	29	MS	MS	5	26
NC3A/6-5	1563	MS	H	3	27
NC3A/6-5	2884	MS	H	1	27
NC3A/6-5	2862	H	H	1	2
NC2-8/10	270	H	H	9	14
NC2-8/10	402	MS	H	9	15
NC2-8/10, NC10-6/15	54	H	H	11	3
NC2-8/16	155	H	H	28	11

NC2-8/16	293	MS	MS	4	14
NC2-8/16	301	H	H	4	0
NC2-8/16	2078	MS	MS	0	24
NC2-8/16	2128	H	H	2	3
NC3A/12	1936	H	H	16	30
NC3A/12	1959	H	H	12	8
NC3A/13	265	H	H	3	6
NC3A/13	1366	H	H	11	4
NC3A/13	2101	MS	H	5	18
NC3A/13	2125	H	H	1	0
NC3A/18	1412	H	H	4	0
NC3A/18	1964	H	H	7	10
NC3A/18	2851	H	H	14	13
NC3A/18	2905	H	H	19	15

Table S3 Sex ratios of BC3 progenies by mother's sex and father. Five fathers were crossed with both MS and H mothers while 6 fathers only with H mothers. P-values (without superscript) indicate significance of differences between observed within family and total (MS mothers) or expected (H mothers, see Table 2 how to calculate expected numbers) H:MS ratios.

Sex of mothers	Father	Sex progeny		
		H	MS	p
H	NC10-6/13	11	8	0.706
MS	NC10-6/13	12	53	0.941
H	NC10-6/15	16	13	0.863
H	NC16-6x12-2/5	30	19	0.283
H	NC2-8/10	15	16	0.563
MS	NC2-8/10	9	15	0.014
H	NC2-8/16	34	14	0.016
MS	NC2-8/16	7	35	0.808
H	NC3A/12	35	39	0.279
H	NC3A/13	15	10	0.519
MS	NC3A/13	5	18	0.651
H	NC3A/17	42	3	0
H	NC3A/18	44	38	0.987
H	NC3A/6-4	46	16	0.001
MS	NC3A/6-4	11	78	0.159
H	NC3A/6-5	1	2	na
**MS**	**Total**	**44**	**199**	**0.080**[a]
**H**	**Total**	**289**	**178**	**0.000**[a]
**H**	**Expected**	**255**	**221**	**0.000**[b]
**H**	**Total**[c]	**201**	**159**	**0.302**[a]
**H**	**Expected**[c]	**215**	**145**	**0.133**[b]

a significance of differences between fathers
b significance of differences between observed and expected total H:MS ratios
c Total and expected numbers when NC3A/17 and NC3A/6-4 families are excluded
