# Supplementary material for: Cytoplasmic Male Sterility Contributes to Hybrid Incompatibility Between Subspecies of Arabidopsis lyrata
Source: G3 (Bethesda). 2013 Oct 1;3(10):1727–40. doi: 10.1534/g3.113.007815 (PMC3789797; doi:10.1534/g3.113.007815)
Supplement: Supporting Information [file supp_g3.113.007815_FigureS2.pdf]

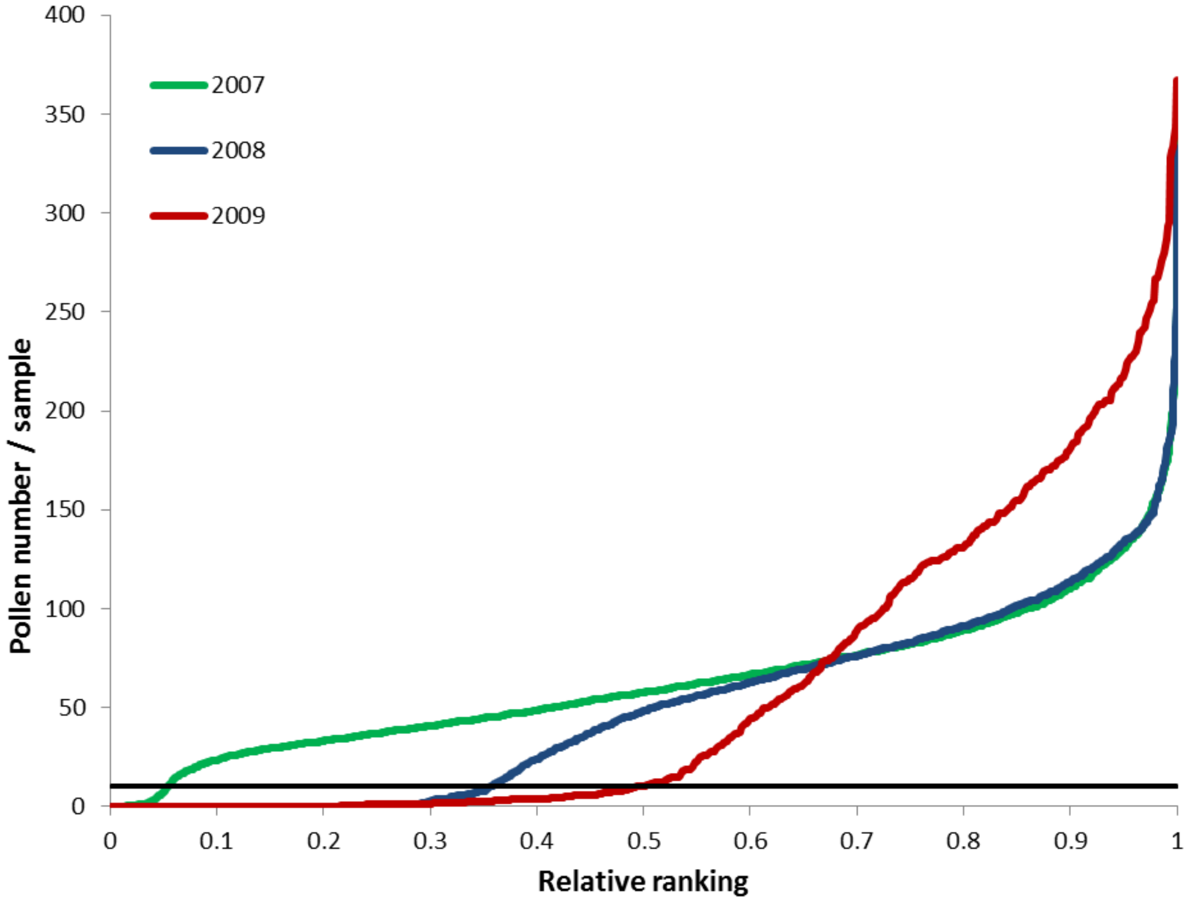

Figure S2 Pollen numbers per sample of the plants in the greenhouse experiments ranked by increasing order. Black vertical line indicates threshold value below which the plants were classified as male sterile.
