# Supplementary material for: Cytoplasmic Male Sterility Contributes to Hybrid Incompatibility Between Subspecies of Arabidopsis lyrata
Source: G3 (Bethesda). 2013 Oct 1;3(10):1727–40. doi: 10.1534/g3.113.007815 (PMC3789797; doi:10.1534/g3.113.007815)
Supplement: Supporting Information [file supp_g3.113.007815_FigureS3.pdf]

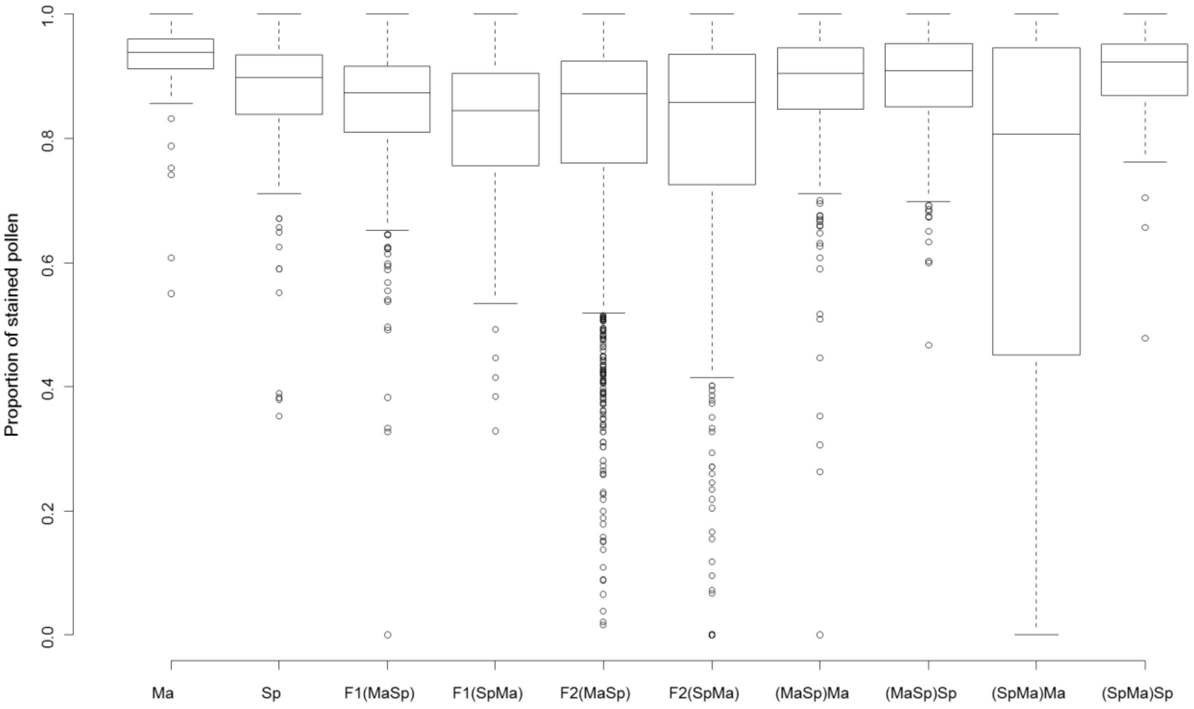


**Figure S3** Pollen viability summary of 2007 experiment. Proportion of good (stained) pollen (horizontal line: median, box: quartiles, dots: outliers) for parental populations, F1 and F2 hybrid reciprocals and four types of backcrosses.
