# Supplementary material for: Cytoplasmic Male Sterility Contributes to Hybrid Incompatibility Between Subspecies of Arabidopsis lyrata
Source: G3 (Bethesda). 2013 Oct 1;3(10):1727–40. doi: 10.1534/g3.113.007815 (PMC3789797; doi:10.1534/g3.113.007815)
Supplement: Supporting Information [file supp_g3.113.007815_FigureS4.pdf]

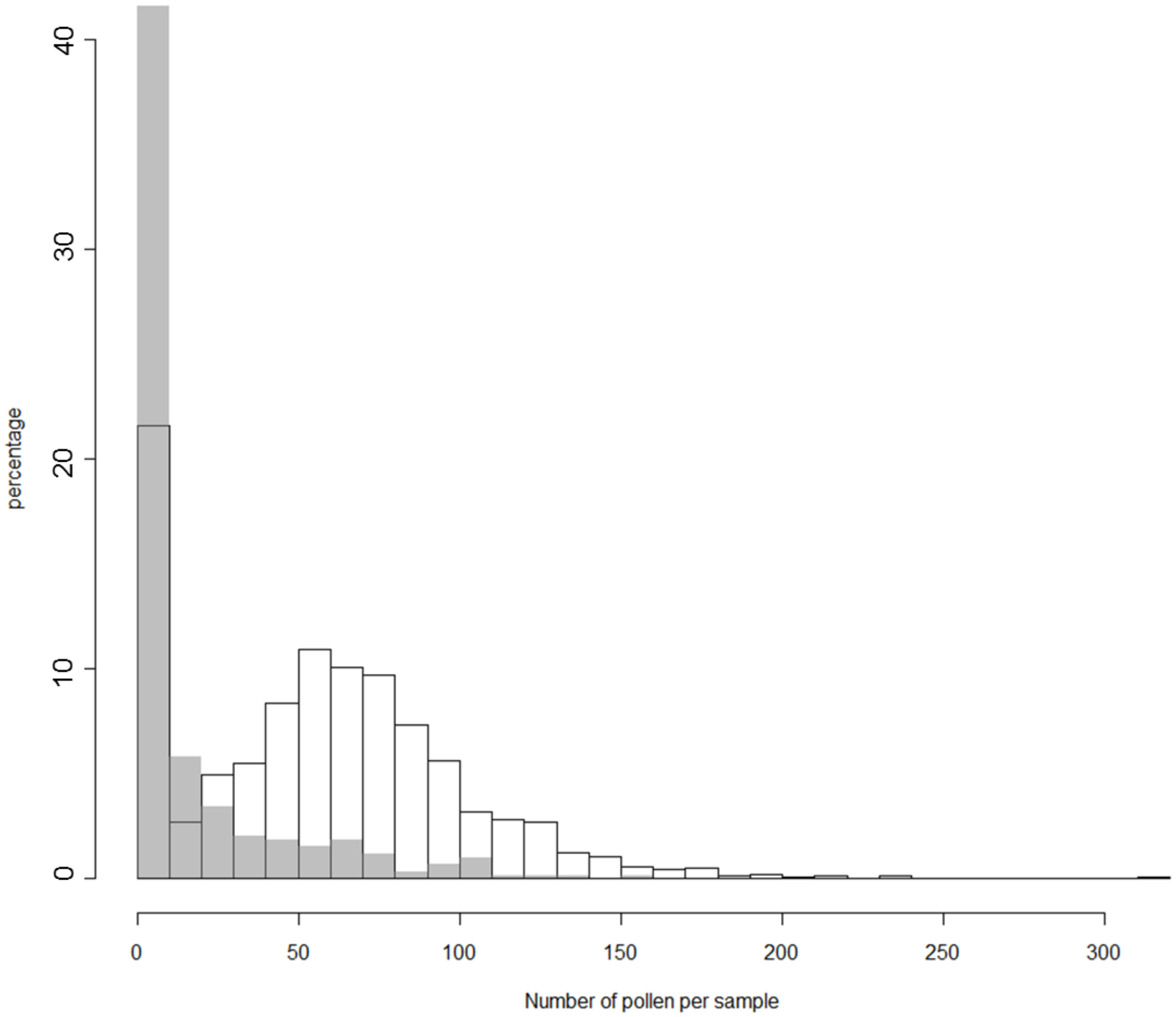

Figure S4 Pollen production of good and bad anthers. Number of pollen per sample produced by plants with poor anthers (grey shading) and plants having normally looking anthers (black borders) in year 2008 experiment.
