# Supplementary material for: Cytoplasmic Male Sterility Contributes to Hybrid Incompatibility Between Subspecies of Arabidopsis lyrata
Source: G3 (Bethesda). 2013 Oct 1;3(10):1727–40. doi: 10.1534/g3.113.007815 (PMC3789797; doi:10.1534/g3.113.007815)
Supplement: Supporting Information [file supp_g3.113.007815_FigureS5.pdf]

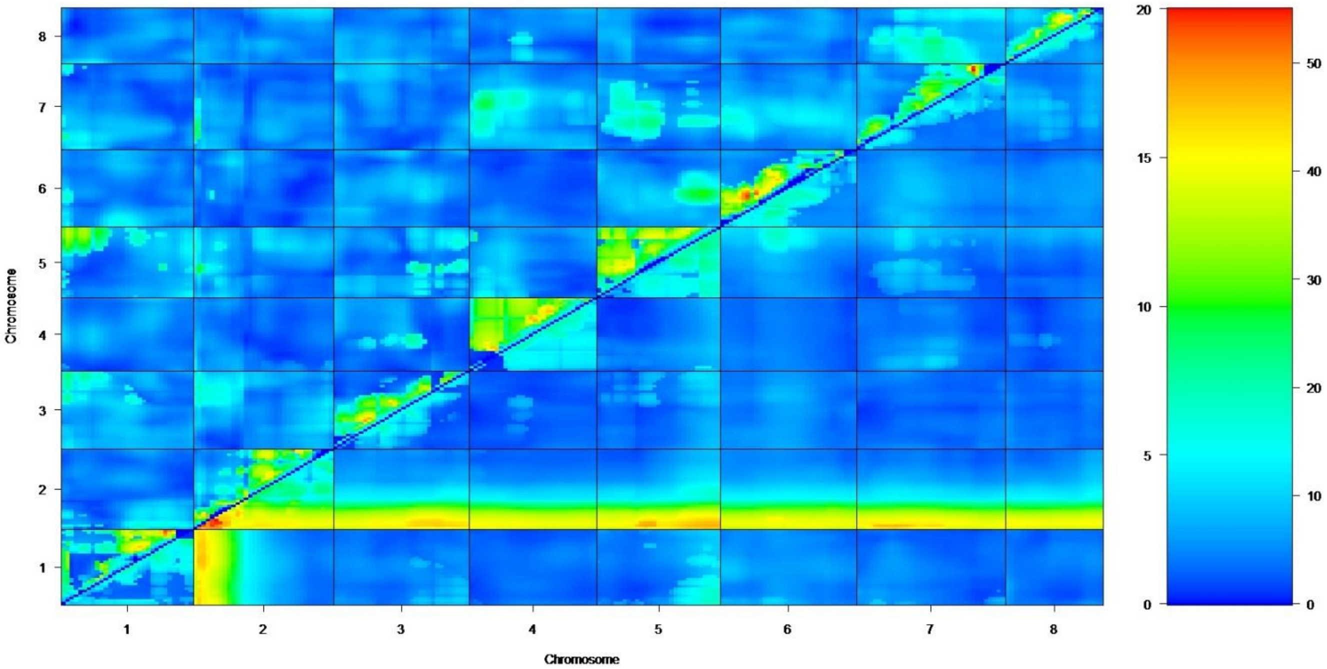

Figure S5 Two QTL interaction scan for *rf* in SpMaF2. The QTL at the beginning of chromosome 2 is clearly visible, but there are not any interactions with it and other loci.
