# Supplementary material for: Cytoplasmic Male Sterility Contributes to Hybrid Incompatibility Between Subspecies of Arabidopsis lyrata
Source: G3 (Bethesda). 2013 Oct 1;3(10):1727–40. doi: 10.1534/g3.113.007815 (PMC3789797; doi:10.1534/g3.113.007815)
Supplement: Supporting Information [file supp_g3.113.007815_FigureS7.pdf]

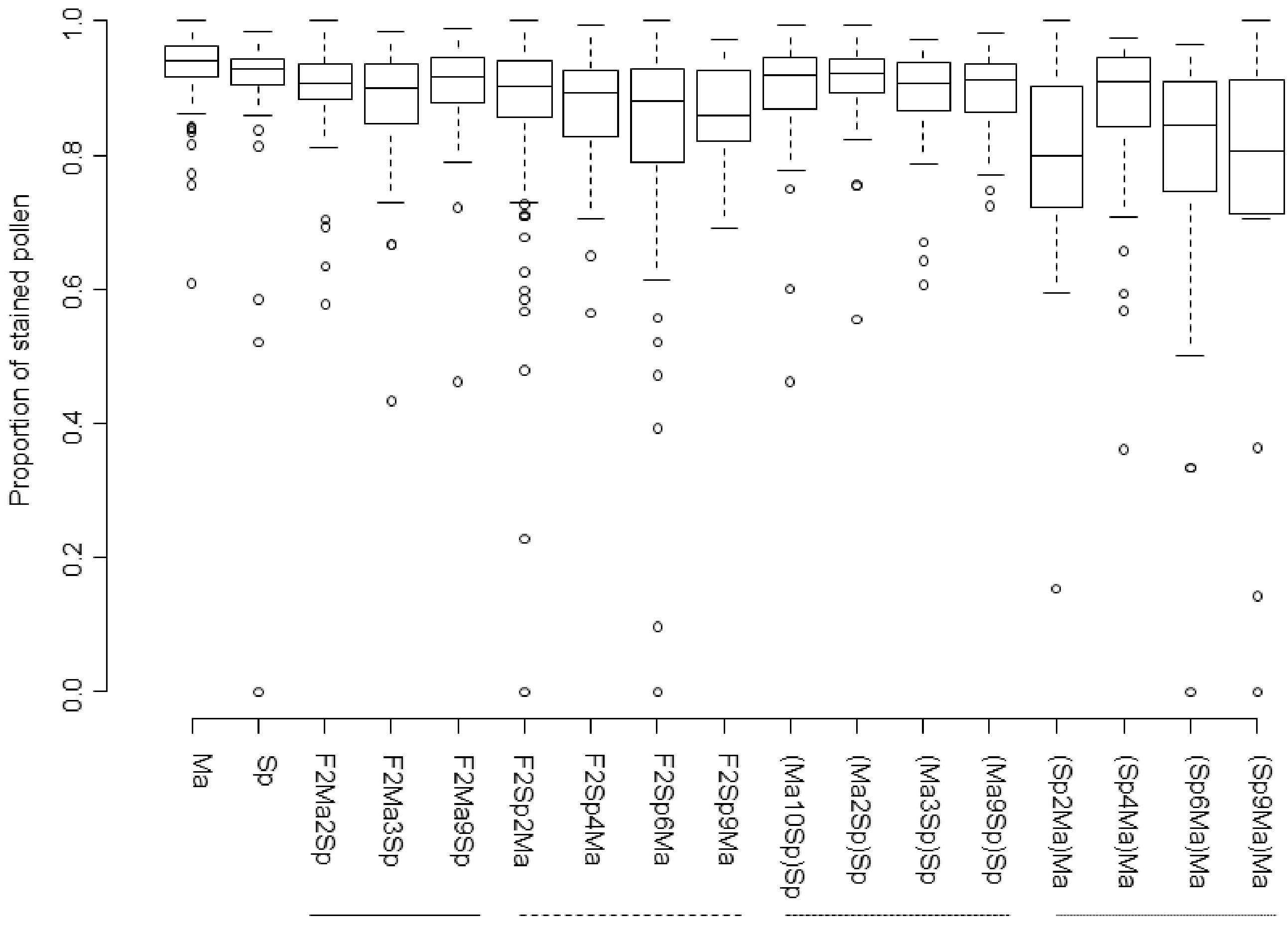


**Figure S7** Pollen viability summary of 2008 experiment. Proportion of good (stained) pollen (horizontal line: median, box: quartiles, dots: outliers) for parental populations, 3 MaSpF2 families (continuous line), 4 SpMaF2 families (dashed line), 4 (MaSp)Sp families (square-dotted line) and 4 (SpMa)Ma families (round-dotted line).
