# Supplementary material for: Cytoplasmic Male Sterility Contributes to Hybrid Incompatibility Between Subspecies of Arabidopsis lyrata
Source: G3 (Bethesda). 2013 Oct 1;3(10):1727–40. doi: 10.1534/g3.113.007815 (PMC3789797; doi:10.1534/g3.113.007815)
Supplement: Supporting Information [file supp_g3.113.007815_FigureS8.pdf]

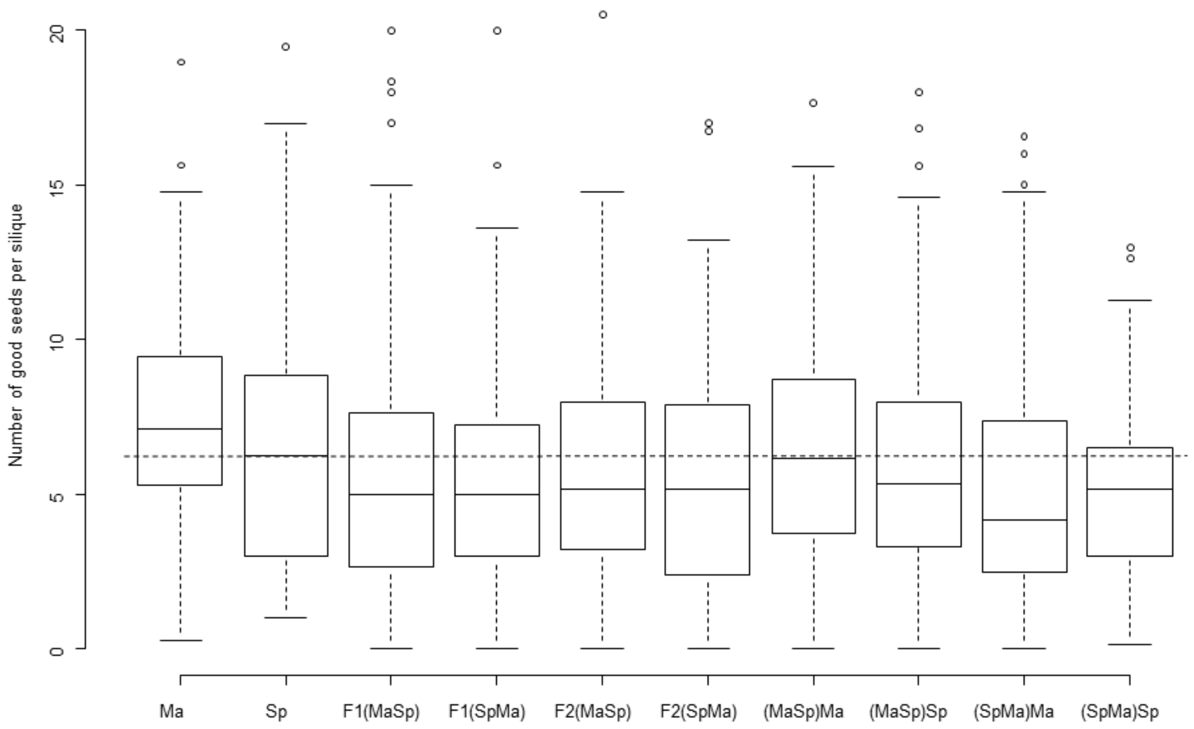

Figure S8 Seed count. Number of good seeds per sample produced by plants in year 2007 experiment (horizontal line: median, box: quartiles, dots: outliers) for parental populations, F1 and F2 hybrid reciprocals and four types of backcrosses. Dashed horizontal line indicates lower parental (Sp) mean seed production. None of the pairwise differences between Sp and the others were significant in Kruskall-Wallis test (total p = 0.00045).
