# Supplementary material for: Cytoplasmic Male Sterility Contributes to Hybrid Incompatibility Between Subspecies of Arabidopsis lyrata
Source: G3 (Bethesda). 2013 Oct 1;3(10):1727–40. doi: 10.1534/g3.113.007815 (PMC3789797; doi:10.1534/g3.113.007815)
Supplement: Supporting Information [file supp_g3.113.007815_FigureS9.pdf]

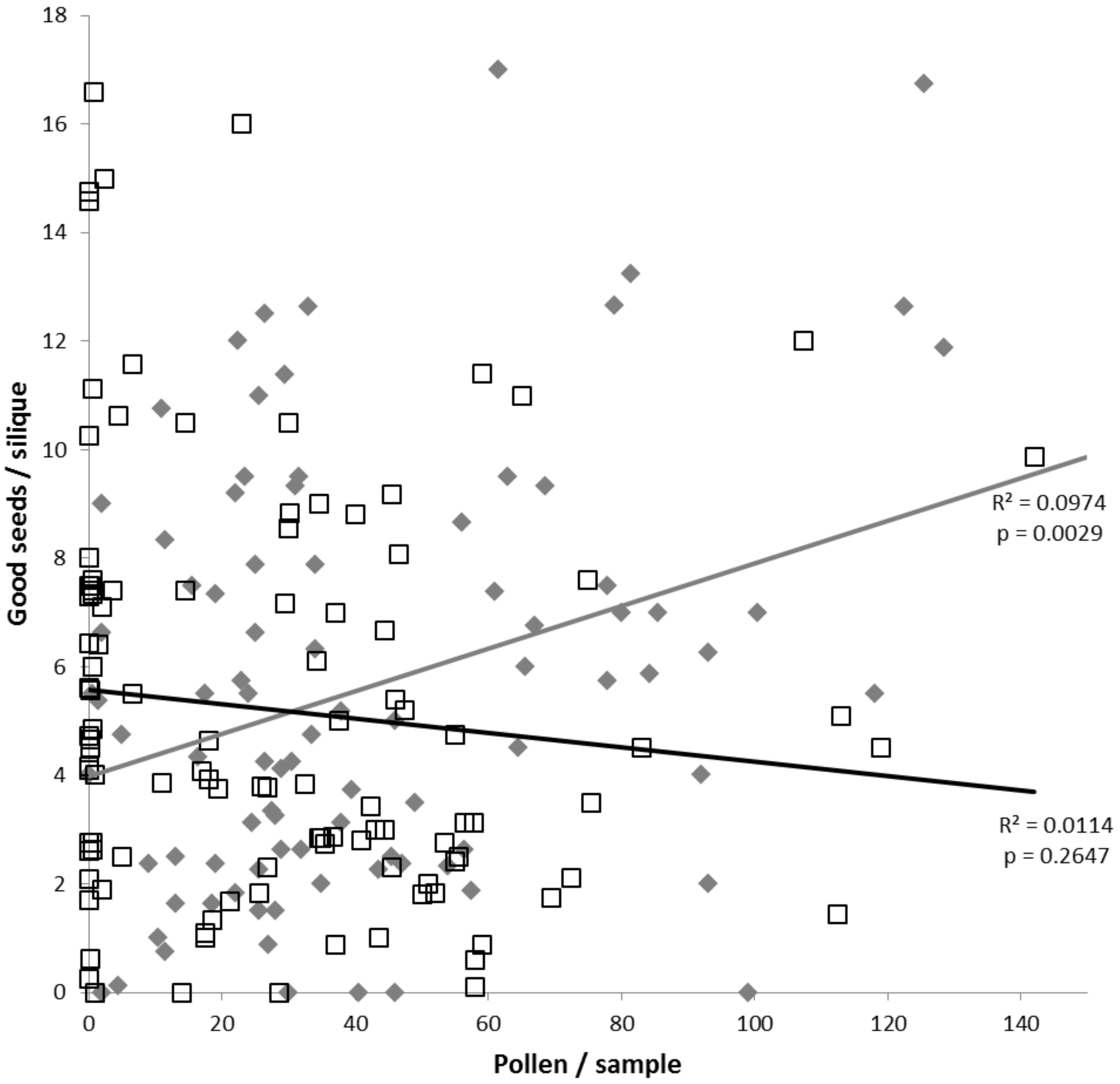

Figure S9 Correlation of seed and pollen production. Male and female fertility of (SpMa)F2 (grey diamonds) are positively correlated, but in (SpMa)Ma backcross plants (open squares) there is no significant correlation.
