# Supplementary material for: Cytoplasmic Male Sterility Contributes to Hybrid Incompatibility Between Subspecies of Arabidopsis lyrata
Source: G3 (Bethesda). 2013 Oct 1;3(10):1727–40. doi: 10.1534/g3.113.007815 (PMC3789797; doi:10.1534/g3.113.007815)
Supplement: Supporting Information [file supp_g3.113.007815_TableS1.pdf]

Table S1 H:MS ratios of BC2 families. Almost all MS BC1 mothers raised mostly MS progeny, but there was more variation in H:MS ratios of H mothers.

Mother	Father	Sex mother	H	MS
SpMaMa14-10	NC22-1	H	5	7
SpMaMa14-4	NC22-1	H	2	1
SpMaMa14-5	NC22-1	H	3	13
SpMaMa14-6	NC22-1	H	8	9
SpMaMa15-1	NC22-1	H	3	7
SpMaMa15-16	NC22-1	H	4	12
SpMaMa15-17	NC22-1	H	3	0
SpMaMa15-8	NC22-1	H	2	0
SpMaMa15-9	NC22-1	H	3	13
SpMaMa1-10	NC22-1	H	32	13
SpMaMa1-16	NC22-1	H	12	5
SpMaMa1-2	NC22-1	H	1	12
SpMaMa1-3	NC22-1	H	2	6
SpMaMa1-5	NC22-1	H	7	6
SpMaMa1-6	NC22-1	H	5	32
SpMaMa1-7	NC22-1	H	4	4
SpMaMa8-11	NC22-1	H	5	0
SpMaMa8-2	NC22-1	H	1	1
SpMaMa8-3	NC22-1	H	9	6
SpMaMa8-7	NC22-1	H	2	2
SpMaMa8-8	NC22-1	H	9	5
SpMaMa8-9	NC22-1	H	5	0

SpMaMa10-1	NC22-1	H	4	11
SpMaMa10-8	NC22-1	H	0	3
SpMaMa11-11	NC22-1	H	3	0
SpMaMa11-14	NC22-1	H	6	1
SpMaMa11-15	NC22-1	H	11	1
SpMaMa11-17	NC22-1	H	4	1
SpMaMa11-18	NC22-1	H	17	0
SpMaMa11-19	NC22-1	H	0	2
SpMaMa11-21	NC22-1	H	12	2
SpMaMa11-4	NC22-1	H	7	2
SpMaMa11-6	NC22-1	H	3	0
SpMaMa11-7	NC22-1	H	3	2
SpMaMa11-8	NC22-1	H	14	0
SpMaMa11-9	NC22-1	H	9	2
SpMaMa16-11	NC22-1	H	2	2
SpMaMa16-2	NC22-1	H	19	7
SpMaMa16-3	NC22-1	H	3	8
SpMaMa16-4	NC22-1	H	12	22
SpMaMa16-5	NC22-1	H	1	2
SpMaMa16-6	NC22-1	H	6	2
SpMaMa16-7	NC22-1	H	21	10
SpMaMa16-7	NC22-1	H	21	10
SpMaMa16-8	NC22-1	H	3	7
SpMaMa9-11	NC22-1	H	26	16
SpMaMa9-17	NC22-1	H	10	10

SpMaMa9-19	NC22-1	H	22	15
SpMaMa9-21	NC22-1	H	10	12
SpMaMa9-22	NC22-1	H	3	2
SpMaMa9-23	NC22-1	H	7	20
SpMaMa9-3	NC22-1	H	14	54
SpMaMa9-4	NC22-1	H	1	1
SpMaMa9-7	NC22-1	H	4	29
SpMaMa9-8	NC22-1	H	23	12
SpMaMa14-5	NC29-2	H	5	4
SpMaMa15-10	NC29-2	H	62	79
SpMaMa1-1	NC29-2	H	6	2
SpMaMa1-12	NC29-2	H	1	1
SpMaMa1-7	NC29-2	H	5	1
SpMaMa11-12	NC29-2	H	4	0
SpMaMa11-7	NC29-2	H	8	6
SpMaMa14-1	NC22-1	MS	1	8
SpMaMa14-2	NC22-1	MS	0	8
SpMaMa14-8	NC22-1	MS	1	28
SpMaMa15-11	NC22-1	MS	0	1
SpMaMa15-13	NC22-1	MS	2	4
SpMaMa15-14	NC22-1	MS	2	3
SpMaMa15-7	NC22-1	MS	0	5
SpMaMa1-13	NC22-1	MS	1	14
SpMaMa8-1	NC22-1	MS	2	5
SpMaMa8-12	NC22-1	MS	1	1

SpMaMa10-5	NC22-1	MS	3	11
SpMaMa10-6	NC22-1	MS	9	0
SpMaMa10-7	NC22-1	MS	0	3
SpMaMa11-10	NC22-1	MS	3	4
SpMaMa11-2	NC22-1	MS	9	9
SpMaMa9-1	NC22-1	MS	4	9
SpMaMa9-13	NC22-1	MS	0	11
SpMaMa9-15	NC22-1	MS	2	7
SpMaMa9-16	NC22-1	MS	1	11
SpMaMa9-18	NC22-1	MS	1	19
SpMaMa9-20	NC22-1	MS	1	2
SpMaMa9-6	NC22-1	MS	2	11
SpMaMa9-9	NC22-1	MS	3	11
SpMaMa14-2	NC29-2	MS	0	3
SpMaMa15-18	NC29-2	MS	1	6
SpMaMa15-5	NC29-2	MS	1	5
