# Supplementary material for: Cytoplasmic Male Sterility Contributes to Hybrid Incompatibility Between Subspecies of Arabidopsis lyrata
Source: G3 (Bethesda). 2013 Oct 1;3(10):1727–40. doi: 10.1534/g3.113.007815 (PMC3789797; doi:10.1534/g3.113.007815)
Supplement: Supporting Information [file supp_g3.113.007815_TableS2.pdf]

Table S2 H:MS ratios of BC3. Fathers NC3A/6-4 and 4NC3A/17 produced excess of H progeny (see table S3).

Father	Mother	Sex mother	Sex grandmother	H	MS
NC3A/6-4	1265	H	H	13	5
NC3A/6-4	1400	H	MS	16	5
NC3A/6-4	1957	H	H	17	6
NC3A/17	51	H	H	18	2
NC3A/17	93	H	H	14	0
NC3A/17	393	H	H	10	1
NC10-6/13	22	H	H	11	8
NC10-6/13	289	MS	MS	2	10
NC10-6/13	476	MS	MS	7	22
NC10-6/13	2077	MS	MS	3	21
NC10-6/15	404	H	H	11	12
NC12, NC3A/12	110	H	H	15	1
NC16-6x12-2/5	2462	H	H	14	10
NC16-6x12-2/5	2925	H	H	8	9
NC3A/6-5	29	MS	MS	5	26
NC3A/6-5	1563	MS	H	3	27
NC3A/6-5	2884	MS	H	1	27
NC3A/6-5	2862	H	H	1	2
NC2-8/10	270	H	H	9	14
NC2-8/10	402	MS	H	9	15
NC2-8/10, NC10-6/15	54	H	H	11	3
NC2-8/16	155	H	H	28	11

NC2-8/16	293	MS	MS	4	14
NC2-8/16	301	H	H	4	0
NC2-8/16	2078	MS	MS	0	24
NC2-8/16	2128	H	H	2	3
NC3A/12	1936	H	H	16	30
NC3A/12	1959	H	H	12	8
NC3A/13	265	H	H	3	6
NC3A/13	1366	H	H	11	4
NC3A/13	2101	MS	H	5	18
NC3A/13	2125	H	H	1	0
NC3A/18	1412	H	H	4	0
NC3A/18	1964	H	H	7	10
NC3A/18	2851	H	H	14	13
NC3A/18	2905	H	H	19	15
