# Supplementary material for: Cytoplasmic Male Sterility Contributes to Hybrid Incompatibility Between Subspecies of Arabidopsis lyrata
Source: G3 (Bethesda). 2013 Oct 1;3(10):1727–40. doi: 10.1534/g3.113.007815 (PMC3789797; doi:10.1534/g3.113.007815)
Supplement: Supporting Information [file supp_g3.113.007815_TableS3.pdf]

Table S3 Sex ratios of BC3 progenies by mother's sex and father. Five fathers were crossed with both MS and H mothers while 6 fathers only with H mothers. P-values (without superscript) indicate significance of differences between observed within family and total (MS mothers) or expected (H mothers, see Table 2 how to calculate expected numbers) H:MS ratios.

Sex of mothers	Father	Sex progeny		
		H	MS	p
H	NC10-6/13	11	8	0.706
MS	NC10-6/13	12	53	0.941
H	NC10-6/15	16	13	0.863
H	NC16-6x12-2/5	30	19	0.283
H	NC2-8/10	15	16	0.563
MS	NC2-8/10	9	15	0.014
H	NC2-8/16	34	14	0.016
MS	NC2-8/16	7	35	0.808
H	NC3A/12	35	39	0.279
H	NC3A/13	15	10	0.519
MS	NC3A/13	5	18	0.651
H	NC3A/17	42	3	0
H	NC3A/18	44	38	0.987
H	NC3A/6-4	46	16	0.001
MS	NC3A/6-4	11	78	0.159
H	NC3A/6-5	1	2	na
**MS**	**Total**	**44**	**199**	**0.080**[a]
**H**	**Total**	**289**	**178**	**0.000**[a]
**H**	**Expected**	**255**	**221**	**0.000**[b]
**H**	**Total**[c]	**201**	**159**	**0.302**[a]
**H**	**Expected**[c]	**215**	**145**	**0.133**[b]

a significance of differences between fathers
b significance of differences between observed and expected total H:MS ratios
c Total and expected numbers when NC3A/17 and NC3A/6-4 families are excluded
