# Supplementary material for: Cytoplasmic Male Sterility Contributes to Hybrid Incompatibility Between Subspecies of Arabidopsis lyrata
Source: G3 (Bethesda). 2013 Oct 1;3(10):1727–40. doi: 10.1534/g3.113.007815 (PMC3789797; doi:10.1534/g3.113.007815)
Supplement: Supporting Information [file supp_g3.113.007815_FigureS1.pdf]

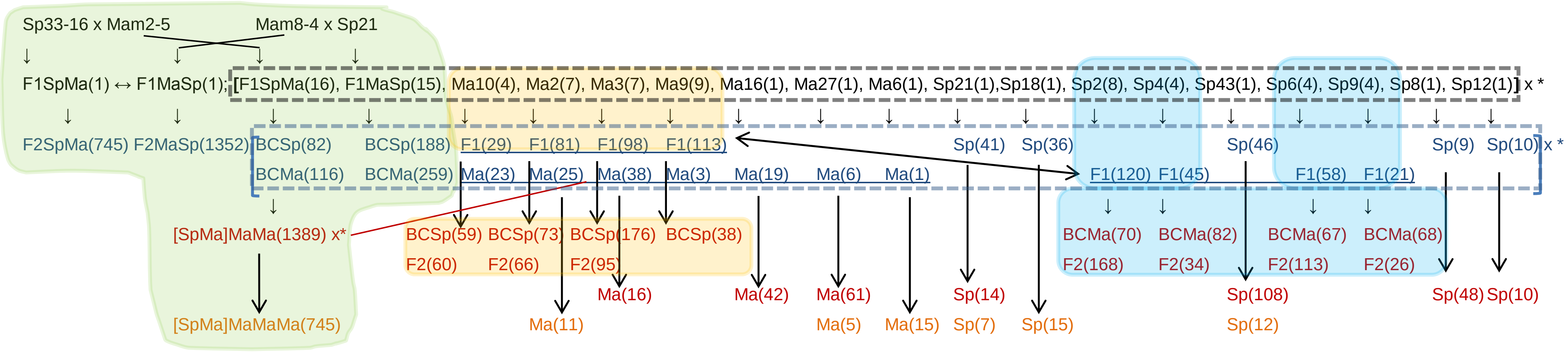

* 2 pollinator plants: Sp38-23, Ma7-10

* 4 pollinator plants: Sp19, Sp11-3, Ma22-1, Ma29-2

* 11 pollinator plants: Ma10-6/13, Ma10-6/15, Ma2-8/10, Ma2-8/16, Ma3A/6-4, Ma3A/6-5, Ma3A/12, Ma3A/13, Ma3A/17, Ma3A/18, Ma16-6x12-2/5

Figure S1 Crossing scheme. Years are indicated by colors: 2005 (or earlier), 2007, 2008, 2009. x = one way cross. ↔ = two way cross. Arrows indicate progeny from the mother plant. Numbers in parentheses indicate number of sister plants grown. Plants surrounded by brackets and dashed boxes are included in same crosses, pollinators of which are below the scheme. Light background colors indicate plants belonging to the same experiment: Green = main crossing family (F2s used in QTL mapping); yellow = four families for studying CMS polymorphism in Ma; blue = four families for studying CMS polymorphism in Sp. BCSp = backcross to Spiterstulen ([MaSp]Sp and [SpMa]Sp), BCMa = backcross to Mayodan ([MaSp]Ma and [SpMa]Ma).
